# Lipid nanoparticles from *L. meyenii* Walp mitigate sepsis through multimodal protein corona formation

**DOI:** 10.1016/j.omtm.2025.101491

**Published:** 2025-05-14

**Authors:** Junsik J. Sung, Jacob R. Shaw, Josie D. Rezende, Shruti Dharmaraj, Andrea L. Cottingham, Mehari M. Weldemariam, Jace W. Jones, Maureen A. Kane, Ryan M. Pearson

**Affiliations:** 1 Department of Pharmaceutical Sciences, University of Maryland School of Pharmacy, 20 N. Pine Street, Baltimore, MD 21201, USA; 2 Department of Microbiology and Immunology, University of Maryland School of Medicine, 685 W. Baltimore Street, Baltimore, MD 21201, USA; 3 Marlene and Stewart Greenebaum Comprehensive Cancer Center, University of Maryland School of Medicine, 22 S. Greene Street, Baltimore, MD 21201, USA

**Keywords:** lipid nanoparticles, inflammation, cytokines, acute phase response, sepsis, protein corona

## Abstract

Plant-derived lipid nanoparticles (PDNPs) are nano-sized particles isolated from various edible plants that contain bioactive components involved in regulating biological responses. Here, we isolated maca-derived lipid nanoparticles (MDNPs) from *Lepidium meyenii* Walp (maca), evaluated their therapeutic effects using two representative lethal models of sepsis, and determined their multimodal anti-inflammatory mechanism that relied on broad sequestration and neutralization of multiple pro-inflammatory cytokines and acute phase proteins (APPs) through formation of a protein corona. Lipidomics of MDNPs revealed triacylglycerols and phytoceramides as major constituents. *In vitro* studies showed that MDNPs were non-toxic, reduced macrophage activation, and sequestered lipopolysaccharide (LPS)-induced pro-inflammatory cytokines, while mitigating nuclear factor kappa B (NF-κB) activity. In a pre-established LPS-induced endotoxemia model, MDNP treatment significantly reduced systemic pro-inflammatory cytokines, reduced organ damage, and increased survival. Untargeted proteomics and bioinformatics analysis identified an enrichment in APPs present in MDNP protein coronas and corresponding inflammatory pathways modulated. The efficacy of MDNPs were further tested using a lethal polymicrobial sepsis model, where treatment significantly improved survival even in the absence of antibiotics. This study identifies MDNPs as an effective strategy capable of inducing potent anti-inflammatory responses, offering significant therapeutic potential for diseases such as sepsis, while informing the future design of synthetic lipid nanoparticles.

## Graphical abstract

**Figure:**
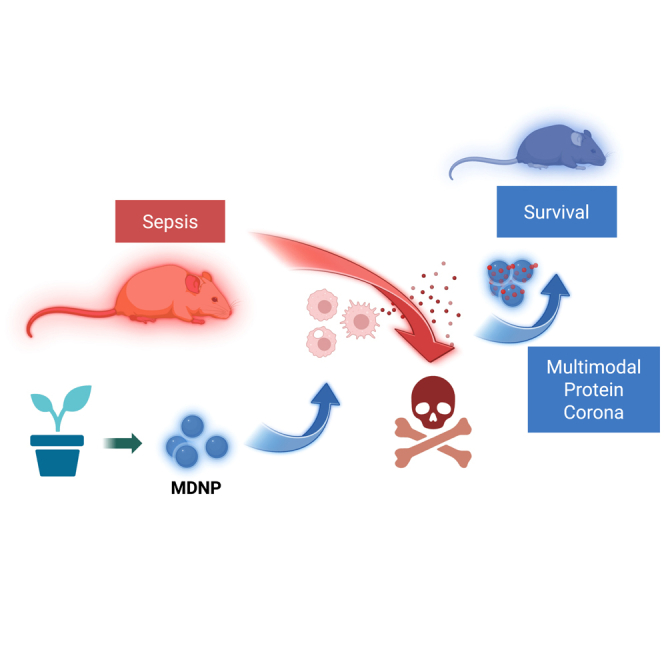

## Teaser

The development of a plant-derived lipid nanoparticle with multimodal capacity to neutralize pro-inflammatory cytokines and acute phase proteins within its protein corona to reduce severe inflammation has vast implications for treating a variety of inflammatory diseases, representing a cost effective, efficient, and valuable advancement in translational medicine.

## Introduction

Edible plant-derived nanoparticles (PDNPs) are nanostructured and membrane-enveloped vesicles secreted by plant cells that serve as carriers of various endogenous bioactive substances. Although previously perceived as cellular debris, PDNPs are recognized as crucial entities that regulate cell-cell communication and immune responses against pathogens.^1^ The evolving understanding of the biogenesis of PDNPs has been described in previous literature.^2^^,^^3^ Briefly, the process initiates with the formation of a *trans*-Golgi network or early endosome. Plant cells then generate the matured multivesicular endosome that integrates and fuses with the plasmalemma, releasing PDNPs through the inward budding of multivesicular endosomes.^4^ There are several studies pointing to the potential therapeutic benefits of PDNPs, which in some cases demonstrate anti-inflammatory,^5^ antioxidant,^6^ and anti-cancer activity.^7^ This emerging class of naturally derived nanoparticles offers highly advantageous features for use as nanomedicines, exhibiting negligible toxicity or immunogenicity, efficient cellular uptake, and having capacity to deliver a variety of therapeutic agents through re-engineering.^8^

Cytokine secretions and the acute phase response (APR) are early, systemic immune reactions that develop within minutes to hours as part of the body’s early defense mechanism to injury, infection, or immune challenge.^9^^,^^10^ The APR is triggered by the release of pro-inflammatory cytokines, such as interleukin-6 (IL-6), tumor necrosis factor alpha (TNF-α), interleukin-1 beta (IL-1β), and others, by macrophages, neutrophils, and various immune cells at the site of inflammation.^11^ This response triggers the production of acute phase proteins (APPs), which are typically undetectable under healthy conditions but increased 10- to 1,000-fold during inflammation.^12^ These proteins play roles in modulating inflammation, enhancing pathogen clearance, and promoting tissue repair.^13^ The APR is generally recognized as beneficial for restoring homeostasis disturbed by such injuries; however, APPs have been described to elicit a range of functions to induce pro- or anti-inflammatory responses. During severe inflammatory responses like sepsis, a dysregulated APR can potentiate the inflammatory response leading to coagulopathy, organ failure, and even death.^9^^,^^14^^,^^15^

Several groups have developed strategies to mitigate the inflammatory response by sequestering pro-inflammatory cytokines.^16^ Multiple studies highlight that the use of membrane-coated nanoparticles that mimic biological cell membranes to trap and neutralize these inflammatory cytokines.^17^ By acting as macrophage decoys, these nanoparticles can be developed to bind and neutralize endotoxins and pro-inflammatory cytokines.^18^ Similarly, neutrophil membrane-coated nanoparticles are produced using neutrophils isolated from mouse bone marrow after lipopolysaccharide (LPS) stimulation.^19^ The process involves membrane extraction, incubation, and extrusion or sonication with polymeric nanoparticle cores. These membrane-camouflaged nanoparticles reduce inflammation by binding and neutralizing endotoxins and cytokines such as IL-6 and TNF-α.^18^^,^^20^ Despite these advancements, the complex synthesis and formulation required for these designs to sequester pro-inflammatory mediators presents a significant challenge to clinical translation. As a result, there is critical need to develop simpler anti-inflammatory strategies that can sequester multiple inflammatory mediators to mitigate pro-inflammatory responses to augment sepsis survival.

*Lepidium meyenii* Walp*,* also referred to as maca, is a biennial root plant indigenous to the Peruvian Andes. Maca possesses high contents of fiber, amino acids, fatty acids, and other essential nutrients, while also containing various bioactive compounds that are involved in integral cellular activities within plants.^21^ Several studies have shown that maca alleviates fatigue,^22^ oxidative stress,^23^ tumor formation,^24^ and inflammation.^25^ However, studies have focused on the raw material or crude extract, which contains mixture of different active ingredients, to evaluate in disease models.^26^^,^^27^ Despite these known advantages, maca remains relatively underexplored.

This investigation demonstrates the isolation, characterization, and therapeutic development of a PDNPs isolated from maca root, termed maca-derived lipid nanoparticles (MDNPs), for the treatment of severe inflammation as exemplified using *in vitro* and *in vivo* models of LPS-induced endotoxemia and polymicrobial sepsis. MDNPs were isolated and characterized for physicochemical properties, as well as lipid composition using lipidomic analysis. The toxicity profile of MDNPs was then established prior to determining their uptake profile and anti-inflammatory properties. *In vitro*, MDNPs efficiently sequestered multiple pro-inflammatory cytokines, which led to comprehensive *in vivo* assessment of their biodistribution and therapeutic activity. Therapeutic administration of MDNPs to LPS-challenged mice led to significant reductions in plasma pro-inflammatory cytokines, reductions in inflammation-induced organ damage, and improved survival. When nanoparticles are introduced into a biological fluid, they are rapidly covered by a layer of biomolecules known as the biomolecular corona (or protein corona).^28^^,^^29^ To identify the mechanism by which MDNPs elicited its anti-inflammatory effects, we performed untargeted proteomics analysis of the MDNP protein corona to uncover the inflammatory mediators sequestered by MDNPs and corresponding pathways and upstream regulators modulated. MDNPs were found to sequester and neutralize a variety of APPs, which promote the propagation of pro-inflammatory immune responses, in addition to pro-inflammatory cytokines. Lastly, the efficacy of MDNPs was assessed using a clinically relevant mouse model of polymicrobial sepsis and found to significantly increase survival. These results demonstrate the potential of MDNPs as an abundant, cost effective, naturally derived therapeutic agent for use as a multimodal intervention for a variety of inflammatory diseases due to their ability to sequester a broad spectrum of inflammatory mediators.

## Results

### Isolation and characterization of MDNPs

**Figure 1:**
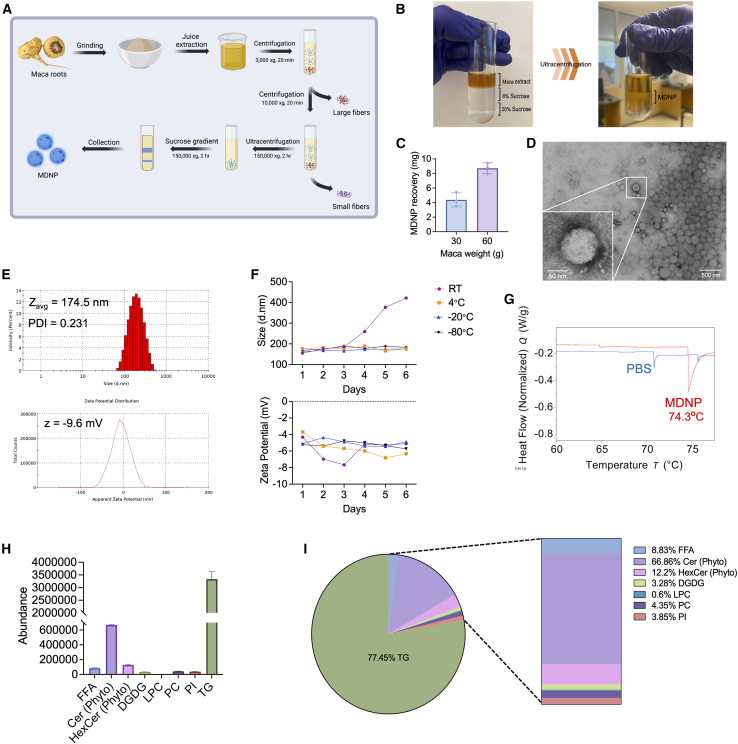
Isolation and characterization of MDNPs (A) Schematic representation of the isolation process for maca-derived lipid nanoparticles (MDNPs). Created in BioRender. Sung, J. (2025) https://BioRender.com/h22o988. (B) Before and after images of MDNP isolation. (C) MDNP recovery from pure maca powder. (D) A representative TEM image of MDNPs. (E) Room temperature dynamic light scattering (DLS) measurement of size (174.5 nm), PDI (0.231), and zeta potential (−9.6 mV) of freshly isolated MDNPs. (F) Storage stability test of MDNPs up to 6 days under various storage temperatures. (G) Heat flow curve of MDNPs measured by differential scanning calorimetry showing the summit of melting peak at 74.3°C. (H) Bar graph showing the abundance of lipids identified after bulk lipid extraction and component analysis by liquid chromatography coupled to high resolution mass spectrometry (LC-MS/MS). (I) Pie graph illustrating the whole ratio of each component, and the vertical slice magnifies less abundant lipid groups. Data is representative of mean ± SD (*n* = 3).

MDNPs were isolated from maca juice using differential ultracentrifugation and density gradient sucrose gradient centrifugation (Figure 1).^30^ MDNPs accumulated at the interface of 20%/35% sucrose gradient, and the recovery was approximately 4 mg per 30 g of maca powder (Figures 1B and 1C). Transmission electron microscopy (TEM) revealed that MDNPs display a spherical morphology, and their core structure was consistent with that of solid lipid nanoparticles (Figure 1D). Size and zeta potential of MDNPs measured an average of 174.5 nm and −9.6 mV, respectively (Figure 1E). We next assessed the stability of MDNPs at multiple temperatures in PBS. MDNPs could tolerate a freeze and thaw cycle and were stable at 4°C for up to 6 days, the maximum length of time tested. All different temperature storage conditions kept MDNPs stable except for room temperature (RT), where after 3 days the size was significantly increased (Figure 1F). The stability of MDNPs were also tested in cell culture medium and mouse plasma at RT and 37°C. There were minimal changes in size and zeta potential (Figure S1). The sizes and zeta potential of MDNPs were also stable upon lyophilization (Figure S2). Differential scanning calorimetry (DSC) analysis determined the summit of melting peak of MDNPs, which was measured as 74.3°C (Figure 1G).

To determine the lipid composition of MDNPs, untargeted high throughput lipidomics was performed. A bar graph (Figure 1H) and a pie/vertical slice chart (Figure 1I) shows the total lipid abundance and ratio in percentage of each component in MDNPs. The lipidomics identified a total of eight individual lipids in four categories including sphingolipids, galactolipids, neutral lipids, and phospholipids (Figure S3). The most prevalent species within the MDNPs were the triglyceride subclass with 51 identifications (77.45%), followed by phytoceramide (Cer [Phyto]) (14.77%), phytohexosylceramide (HexCer [Phyto]) (2.7%), free fatty acids (FFAs) (1.95%), digalactosyldiacylglycerol (DGDG) (0.72%), phosphatidylcholine (PC) (0.96%), phosphatidylinositol (PI) (0.85%), and lysophosphatidylcholine (LPC) (0.13%) (Table S1).

The complete protein compositional analysis of MDNPs using ultra-high performance liquid chromatrography-tandem mass spectrometry (UPLC-MS/MS) revealed undetectable levels of proteins that could be linked to plant databases from UniProt and the Plant Proteome Database. This aligns with our lipidomics results indicating that MDNPs are primarily composed of lipids, with minimal or no associated protein content. Overall, these data demonstrate the successful isolation, purification, and characterization of MDNPs from bulk maca extract.

### *In vitro* cytotoxicity, internalization, and therapeutic effect of MDNPs

**Figure 2:**
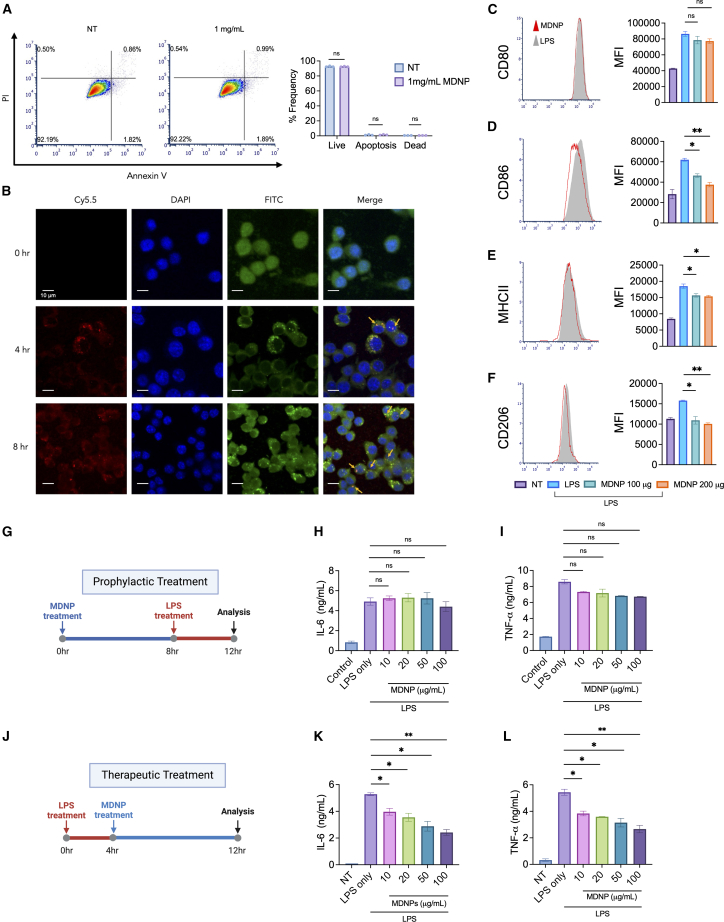
*In vitro* cytotoxicity, cellular internalization, immunomodulation, and therapeutic effects against LPS-induced inflammation (A) PI/annexin V assay by flow cytometry using 1 mg/mL of MDNPs in BMDMs. Quantitative analysis of live, apoptotic, and dead cells after MDNP treatment. (B) Uptake of Cy5.5-labeled MDNPs by BMDMs after 4 and 8 h. Cells were stained with DAPI and FITC-phalloidin to visualize the nucleus and F-actin, respectively. (C–F) Immunomodulatory activity of MDNPs in LPS stimulated BMDMs using flow cytometry of CD80, CD86, MHC II, and CD206 markers. (G) A timeline for prophylactic treatment of MDNPs. Created in BioRender. Sung, J. (2025) https://BioRender.com/x40h804. (H and I) ELISA cytokine measurements of (H) IL-6 and (I) TNF-α. (J) A timeline for therapeutic treatment. (K and L) ELISA cytokine measurements of (K) IL-6 and (L) TNF-α. Data are expressed as mean ± SD (*n* = 3). ∗*p* < 0.05 and ∗∗*p* < 0.01 versus LPS control group.

To analyze potential toxicity, bone marrow-derived macrophages (BMDMs) were treated with MDNPs (1 mg/mL) for 8 h prior to flow cytometry analysis to measure viability and apoptosis (Figure 2A). MDNP treatment did not affect the cell viability or induce apoptosis. Furthermore, we observed a time-dependent cellular uptake of Cy5.5-labeled MDNPs using BMDMs (Figure 2B). The quantitative measurement of mean fluorescence intensity (MFI), a commonly used method in confocal microscopy analysis,^31^ can be found in Figure S4. These findings demonstrate that MDNPs are non-toxic and efficiently internalized by BMDMs.

BMDMs were treated with LPS followed by MDNPs to assess their ability to mitigate immune activation. The observed decrease in CD80 (reduced but not significant), CD86, and major histocompatibility complex (MHC) II expression following MDNP treatment suggests that MDNPs reduced macrophage activation, contributing to a decreased pro-inflammatory response (Figures 2C–2E). Modulation of macrophage polarization is one of the strategies to modulate inflammation, support tissue regeneration, and reestablish disrupted homeostasis.^32^ We also measured a reduction in CD206 as a marker of anti-inflammatory M2-like macrophages, suggesting that MDNPs induced a shift in macrophage phenotype toward a more undifferentiated or M0-like state (Figure 2F). These results indicate that MDNPs can impact the activation status of macrophages, suggesting that they aid in shifting their profile toward a more balanced and neutral immune response.

To assess the anti-inflammatory effects of MDNPs, we investigated its prophylactic and therapeutic activity by treating MDNPs on LPS-challenged BMDMs. In the prophylactic setting, MDNPs were treated for 8 h prior to 4 h LPS exposure (Figure 2G). However, treatment did not significantly attenuate the release of pro-inflammatory cytokines (IL-6 and TNF-α) (Figures 2H and 2I), suggesting that it was unlikely that MDNP treatment directly modulated pro-inflammatory signaling pathways in macrophages responsible for cytokine secretion. In the therapeutic setting, BMDMs were challenged with LPS for 4 h prior to the treatment of MDNPs for 8 h (Figure 2J). The result shows that pro-inflammatory cytokines including IL-6 and TNF-α induced by LPS were significantly reduced, whereas IL-1β and interferon (IFN)-γ were only slightly reduced (Figure S5). Furthermore, additional testing was conducted on the crude extract after MDNP isolation to determine if it possesses any anti-inflammatory efficacy; however, no cytokine reduction was detected (Figure S6). These results suggested that MDNPs can reduce pro-inflammatory responses, and the anti-inflammatory component of maca is the purified MDNPs.

### Sequestration of cytokines and mitigation of NF-κB activation

**Figure 3:**
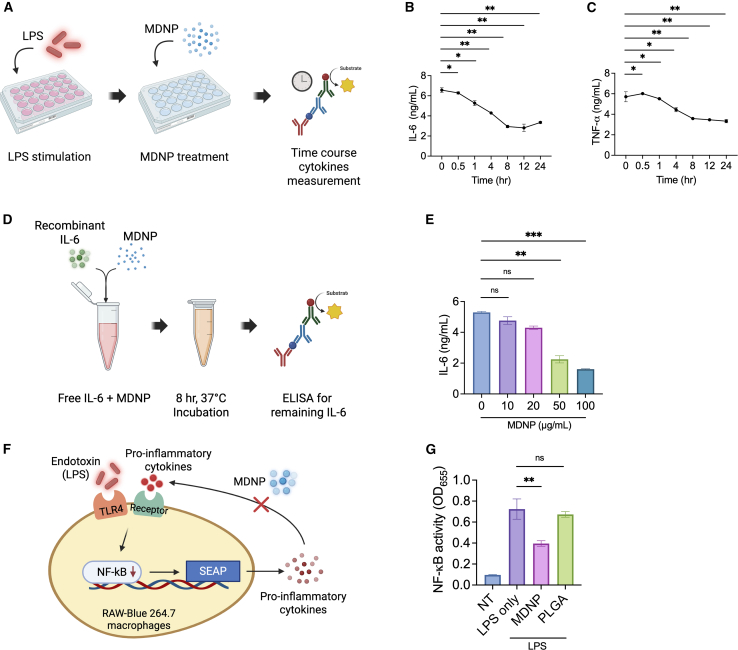
*In vitro* sequestration of pro-inflammatory cytokines from LPS-stimulated BMDMs (A) Schematic representation of LPS stimulation and pro-inflammatory cytokine reduction over time after MDNP treatment. Created in BioRender. Sung, J. (2025) https://BioRender.com/n91r920. (B and C) ELISA cytokine measurements of (B) IL-6 and (C) TNF-α. (D) Schematic of IL-6 cytokine sequestration study. Created in BioRender. Sung, J. (2025) https://BioRender.com/n10c223. (E) Concentration-dependent sequestration of IL-6 protein by MDNPs. (F) Schematic of NF-κB activity assay using RAW-Blue 264.7 cells. Created in BioRender. Sung, J. (2025) https://BioRender.com/y73h708. (G) NF-κB activity after treatment with MDNPs in LPS stimulated cells. PLGA was used as a control. All data are expressed as means ± SD (*n* = 5). ∗∗*p* < 0.01, ∗∗∗*p* < 0.001, and ∗∗∗∗*p* < 0.0001 versus LPS or NT control group.

IL-6 and TNF-α are well known to be involved in pathogenesis of chronic inflammation, autoimmune diseases, and cancer.^33^^,^^34^ Utilizing data from the *in vitro* anti-inflammatory assay, an investigation was conducted to confirm whether MDNPs possess the capability to sequester pro-inflammatory cytokines in the absence of cells. In a time-dependent experiment, BMDMs were treated with 100 ng/mL LPS for 4 h, then the supernatant rich in inflammatory cytokines was incubated with 100 μg/mL MDNPs for up to 24 h (Figure 3A). ELISA analysis of these supernatants indicated a gradual decrease in IL-6 levels, with a maximum of 48% reduction after 8 h (Figure 3B). A similar trend was observed for TNF-α levels, with a 28% reduction over the same time frame (Figure 3C). Optimization of MDNP concentration revealed concentration-dependent cytokine sequestration properties of MDNPs, and 100 μg/mL MDNPs exhibited the most significant sequestration of IL-6 and TNF-α, whereas poly(lactic-co-glycolic acid) (PLGA) nanoparticles (as control) did not display any cytokine sequestration properties (Figure S7). We also confirmed the ability of MDNPs to sequester recombinant IL-6 to exclude the possibility of other proteins contributing to cytokine removal *in vitro* (Figures 3D and 3E). Lastly, to assess whether the cytokine-sequestering ability of MDNPs is dependent on its structural integrity, we solubilized MDNPs in a chloroform:methanol mixture and reassembled it using the thin-film rehydration method, with or without PEGylated lipid or cholesterol (Figure S8). The restructured MDNPs retained its cytokine-binding activity, suggesting that its core properties are essential for function. However, the inclusion of PEGylated lipid abolished cytokine sequestration, while the addition of up to 20% cholesterol impaired this ability, although cytokine levels remained significantly lower than in the LPS control. These results indicate that MDNPs can effectively sequester pro-inflammatory cytokines to mitigate inflammation, and that structural modifications can diminish this function, underscoring the importance of the native MDNP composition.

The innate immune responses that are triggered by LPS are mediated by Toll-like receptor (TLR)-4 and subsequent activation of the transcription factors NF-κB and activator protein 1.^35^ Upon encountering various PAMPs and DAMPs, macrophages undergo rapid activation and secrete a diverse range of cytokines and chemokines including IL-6 and TNF-α.^36^ In this experiment, RAW-Blue cells, an NF-κB macrophage reporter cell line that releases secreted embryonic alkaline phosphatase (SEAP) after activation, were used to gauge MDNP-mediated effects following LPS stimulation (Figure 3F). Following treatment with 100 μg/mL MDNPs for 1 h, a significant decrease in NF-κB activity was measured, confirming that MDNPs possess the capacity to mitigate pro-inflammatory cell signaling and reduce macrophage activation (Figure 3G). PLGA was used as a control, which was unable to reduce NF-κB activation.

### *In vivo* biodistribution and anti-inflammatory effects of MDNPs

**Figure 4:**
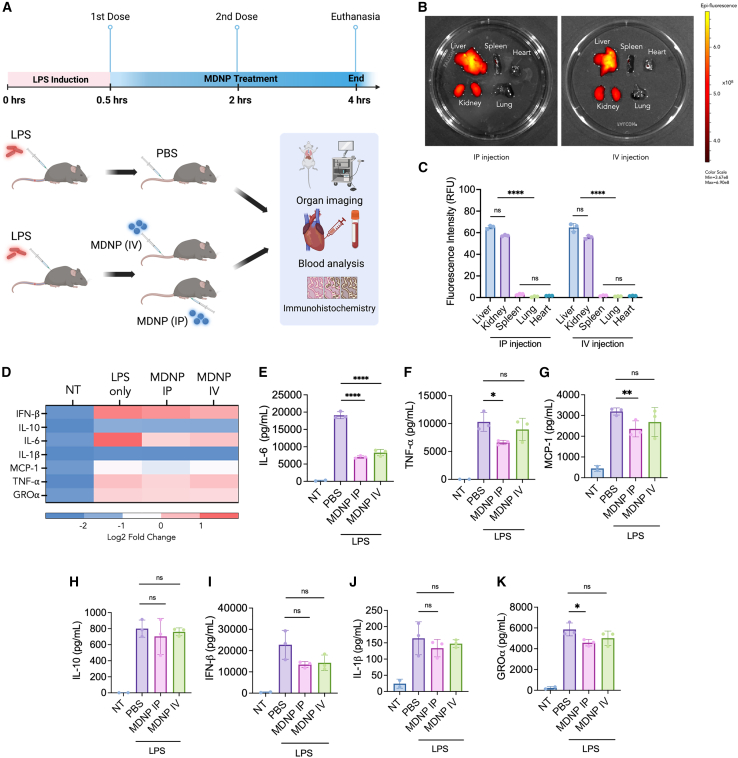
Route of administration-dependent biodistribution and anti-inflammatory effects of MDNPs (A) Timeline of MDNP treatments following LPS challenge. The schematic illustrates i.p. and i.v. injections performed on C57BL/6J mice (LPS only, LPS + i.p. injection, and LPS + i.v. injection, *n* = 3 per group). Created in BioRender. Sung, J. (2025) https://BioRender.com/n92o995. Created in BioRender. Sung, J. (2025) https://BioRender.com/v94b216. (B) IVIS image of biodistribution of fluorescently labeled MDNPs in mouse organs (liver, kidney, spleen, lung, and heart). (C) Fluorescence intensity measurement of MDNPs biodistribution. (D–K) (D) Heatmap of cytokine expression including the following: (E) IL-6, (F) TNF-α, (G) MCP-1, (H) IL-10, (I) IL-1β, (J) IFN-β, and (K) GROα quantification from mice plasma samples. Data are expressed as mean ± SD (*n* = 3). ∗*p* < 0.05, ∗∗*p* < 0.01, ∗∗∗*p* < 0.001, and ∗∗∗∗*p* < 0.0001 versus PBS control group.

For the *in vivo* biodistribution study, MDNPs were labeled with Cy5.5 and administered intraperitoneally (i.p.) or intravenously (i.v.) into LPS-challenged mice (5 mg/kg LPS dose) twice at an MDNP dose of 2 mg/injection at 0.5 and 2 h (Figure 4A). At 4 h, mice were euthanized to image the biodistribution of Cy5.5-MDNPs in different organs including liver, spleen, heart, kidney, and lung with an *in vivo* imaging system (IVIS). Most of the Cy5.5-labeled MDNPs were delivered to the liver and kidney by both i.p. and i.v. injection, and very low amounts were detected in the spleen, heart, and lung (Figures 4B and 4C).

We also investigated how the route of administration (i.p. versus i.v.) affected the *in vivo* therapeutic effects in mice. Mice were challenged with 5 mg/kg LPS i.p., followed by treatment with 2 mg MDNPs at 0.5 h and 2 h post-LPS treatment. After 4 h, mice were euthanized to collect organs samples and plasma (Figure 4A). Plasma samples were processed using a MAGPIX Luminex multiplexing immunoassay system. This system was employed to analyze the expression level of a 7-plex panel of cytokines which included IL-6, TNF-α, MCP-1, IL-10, IL-1β, IFN-β, and growth regulated protein α (GROα). The heatmap demonstrates overall reduction of pro-inflammatory cytokines for both routes (Figure 4D). The analysis revealed a significant reduction in IL-6 level following both administration routes (Figure 4E), and a notable reduction in TNF-α, MCP-1, and GROα for i.p.-administered MDNPs (Figures 4F–4K). Administration of MDNPs by i.v. route also reduced systemic cytokine levels; however it was slightly less effective. We further evaluated blood biochemistry of albumin, alanine transaminase, creatine, aspartate transferase, globulin, total protein (TP), and blood urea nitrogen (BUN). Although minor reductions were observed for MDNP-treated mice, there was no significant alteration in levels of blood biochemistry parameters compared to LPS-treated controls (Figure S9). The non-significant reductions in blood biochemistry following MDNP-treatment could be due to the pre-established severe inflammatory response, which could not be completely reversed by the endpoint of the study.

### Treatment of MDNPs accelerated organ recovery and improved survival

**Figure 5:**
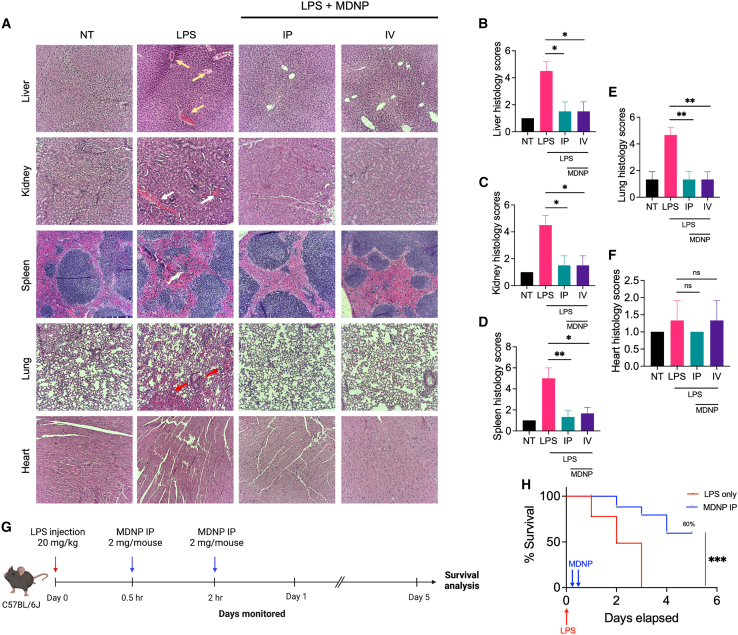
Representative images of organ recovery and survival effect by treatment of MDNPs during LPS-induced sepsis (A) Hematoxylin and eosin (H&E) staining of the liver, kidney, spleen, lung, and heart. The LPS-treated group images shows presence of neutrophil infiltration (yellow arrow) in the liver, tubular necrosis (white arrow) in the tubules in kidney, focal necrosis and dysregulation in white pulp of spleen, appearance of extensive clots (red arrow) in the alveoli in lungs, while no noticeable damage in heart. On the contrary, mice organs administered with i.p. and i.v. displayed notably reduced organ damage similar to the level of the non-treated control group. (B–F) Quantification of H&E staining. Levels of injury scores were calculated. Data are expressed as mean ± SD (*n* = 3). ∗*p* < 0.05 and ∗∗*p* < 0.01 versus LPS control group. (G) Schematic of survival study of LPS-treated and LPS + MDNP-treated C57BL/6J mice (*n* = 10/group). Created in BioRender. Sung, J. (2025) https://BioRender.com/l63d302. (H) The survival analysis graph displays a significant difference between LPS-only and the MDNP-treatment group. Mice were challenged with 20 mg/kg LPS i.p. followed by two injections of MDNPs. Kaplan-Meier curves and a log rank test was performed to compare the survival rates. ∗∗∗*p* < 0.001.

Histological analysis was used to evaluate the level of inflammation in tissues isolated from experiments conducted in Figure 4. H&E stainings of five different organs including the liver, kidney, spleen, lung, and heart are shown in Figure 5. LPS-treated liver showed severe immune cell infiltration, tubular necrosis was detected in the kidney, focal necrosis, and dysregulation in white pulp of spleen was observed, and extensive clots in the alveoli of lung appeared, but no significance of heart injury was measured. On the contrary, the i.v. and i.p. administered MDNP groups displayed notable improvements in histology scores when compared to the LPS group (Figures 5B–5F). Based on the overall safety profile and reductions in pro-inflammatory cytokines, MDNPs were used to further evaluate survival *in vivo*. Mice were challenged with lethal dose of 20 mg/kg LPS in sterile PBS i.p. Then, mice were monitored after two doses of 2 mg MDNPs (Figure 5G). As a result, 60% of MDNP-treated mice survived, whereas 0% of the LPS-treated (control) mice survived (Figure 5H).

### Proteomic profiling of MDNP protein corona

**Figure 6:**
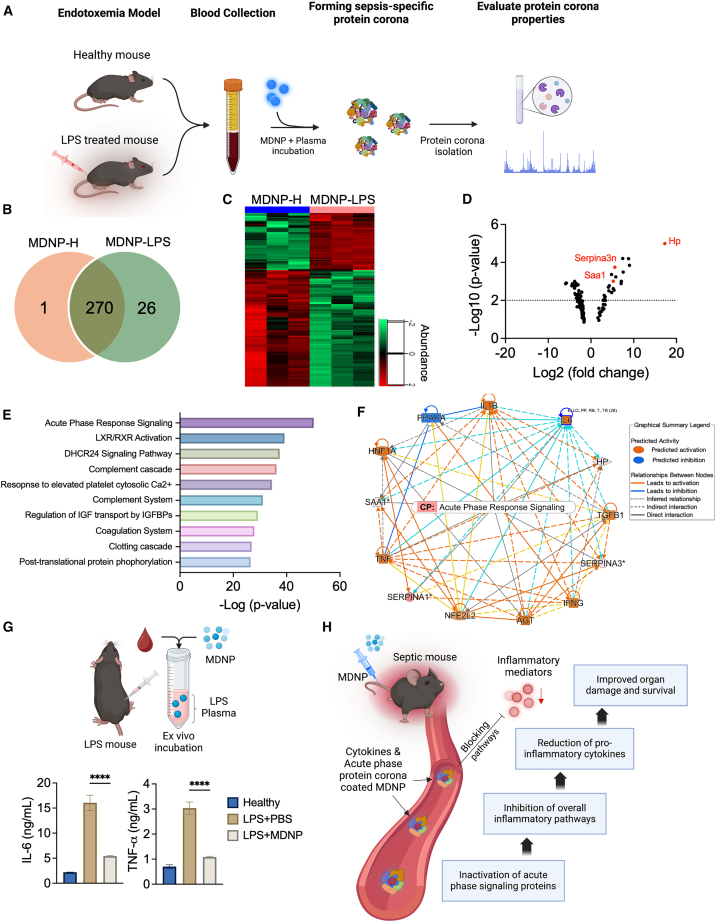
Mapping and identification of protein corona adsorbed onto the surface of MDNPs (A) Schematic showing the process of evaluating the MDNP protein corona. Created in BioRender. Sung, J. (2025) https://BioRender.com/j58w712. (B) Venn diagram illustrating a total of 296 protein corona including unique (1 in healthy and 26 in LPS) and 270 shared proteins in the corona. (C) Heatmap shows abundance of 49 upregulated and 100 downregulated proteins in the LPS compared to healthy MDNP corona groups. (D) Volcano plot of significantly upregulated and downregulated protein corona. Hp, Serpina3n, and Saa1 (red dots) are categorized as pro-inflammatory and acute phase response signaling proteins. (E) Top 10 canonical pathways associated with the significantly enriched corona proteins were identified, with acute phase response signaling being the most prominent pathway. (F) A network representation of acute phase response signaling proteins and major upstream regulators. (G) *Ex vivo* sequestration of pro-inflammatory cytokines by incubating LPS plasma with MDNPs. Both IL-6 and TNF-α was significantly reduced following 8 h incubation with MDNPs. ∗∗∗∗*p* < 0.0001 versus LPS control group. Created in BioRender. Sung, J. (2025) https://BioRender.com/f87s608. (H) A graphical depiction illustrating the proposed mechanism of how the MDNPs sequester and deactivates pro-inflammatory cytokines and acute phase proteins to reduce overall inflammation to enhance sepsis survival. Created in BioRender. Sung, J. (2025) https://BioRender.com/p54m386.

The ability of MDNPs to reduce systemic cytokines and promote LPS mouse survival prompted an exploration of the composition and types of proteins sequestered by the MDNPs. Adsorbed proteins were characterized by forming a protein corona on MDNPs *ex vivo* by incubating with either healthy or LPS-treated mouse plasma (MDNP-H or MDNP-LPS, respectively) (Figure 6A). Proteomics analysis of these coronas identified 297 total proteins (Tables S2 and S3), with 270 common between the two conditions, 1 distinctive for healthy, and 26 for LPS-treated (Figure 6B). Table 1 presents a list of uniquely adsorbed proteins from each group. Among these, CD14, LCN2 (lipocalin-2), APCS (serum amyloid P component), fatty acid binding protein 4, neutrophilic granule protein (NGP), and HSP90AA (heat shock protein 90 alpha) are known to play roles in inflammation and categorized as APPs excluding NGP. The heatmap in Figure 6C shows the differential expression of total 297 detected proteins. Comparing MDNP-H to MDNP-LPS, highlighted significant changes in protein abundance, with 49 proteins being significantly upregulated and 100 proteins downregulated (Figure 6D). Haptoglobin (Hp), which is known for its pro-inflammatory properties,^37^ was found to be the most differentially abundant protein in the MDNP-LPS corona. The top 25 proteins identified in the MDNP-LPS group are shown in Figure S10. Among these, Hp, Seprina3n, and Saa1 are well known for their pro-inflammatory roles in regulating inflammatory process and promoting the release of inflammation cytokines. Their levels can increase dramatically during inflammation.^38^ Figure 6E lists the canonical pathways of MDNP-LPS coronas from ingenuity pathway analysis (IPA). APR signaling was most highly associated with MDNP-LPS corona. The network diagram in Figure 6F illustrates the complex but close interaction of APPs and these upstream pro-inflammatory regulators within the APR signaling pathway. The highlighted connections between APR shows its direct interaction with the APPs sequestered on MDNP-LPS coronas (Hp, Saa1, and SerpinA3N) and a subset of upstream regulators (IL-6, TNF, IL1B, angiotensinogen (AGT), and HNF1A). Importantly, IL-6, TNF, and IL1B play pivotal roles in either indirectly or directly activating these APPs. Figure 6G illustrates the result of *ex vivo* cytokine sequestration assays using MDNPs. It demonstrates that MDNPs retains the ability to bind and remove pro-inflammatory cytokines, such as IL-6 and TNF-α, from the LPS plasma. It is also important to point out that protein corona formed on the surface of MDNPs did not activate any pro-inflammatory responses when treated on BMDMs, supporting that the formation of the corona effectively neutralizes the pro-inflammatory responses of these various mediators (Figure S11). Figure 6H summarizes the proposed mechanism of MDNPs by forming a multimodal protein corona composed of pro-inflammatory cytokines and APPs and neutralization. This inhibits the key inflammatory pathways, blocking the production of pro-inflammatory upstream regulators, ultimately reducing pro-inflammatory cytokines, improving organ function, and increasing survival.

**Table 1:** List of unique proteins adsorbed onto the surface of MDNPs from LPS and healthy plasma

Protein corona	Accession	Description	Gene symbol	Function	Acute phase
LPS plasma	Q8BMT4	transforming growth factor β activator LRRC33	Nrros	key regulator of TGFB1 required for microglia function in nervous system	
	Q9Z1T2	thrombospondin-4	Thbs4	adhesive glycoprotein that mediates cell-to-cell interaction	
	P39876	metalloproteinase inhibitor 3	Timp3	regulates hematopoietic stem cell proliferation, differentiation, and trafficking	
	P02089	hemoglobin subunit beta-2	Hbb-b2	oxygen, CO_2_ transport, and pH regulation	
	P62806	histone H4	H4	central role in transcription regulation, DNA repair, and replication	
	P10810	monocyte differentiation antigen CD14	Cd14	co-receptor for TLR4, and recognize LPS and PAMPs	O
	P11672	neutrophil gelatinase-associated lipocalin	Lcn2	regulates neutrophil recruitment and modulates pro-inflammatory cytokines	O
	P14152	malate dehydrogenase, cytoplasmic	Mdh1	involved in Krebs cycle and plays critical role in cellular energy production	
	P12246	serum amyloid P-component	Apcs	acute-phase reactant that bind to complement proteins and damaged cell	O
	P04117	fatty acid-binding protein, adipocyte	Fabp4	regulates cholesterol ester accumulation and inflammatory responses	O
	P32848	parvalbumin alpha	Pvalb	calcium-binding protein involved in regulation of Ca^2+^ levels in neurons to muscle	
	P70296	phosphatidylethanolamine-binding protein 1	Pebp1	regulate the trafficking and distribution of phosphatidylethanolamine within cells	
	Q99PT1	Rho GDP-dissociation inhibitor 1	Arhgdia	inhibits GDP/GTP exchange activity of Rho GTPases	
	O08692	neutrophilic granule protein	Ngp	enzymatic activity that breaks down bacterial wall that damaged bacterial DNA	
	Q64339	ubiquitin-like protein ISG15	Isg15	antiviral defense by degradation viral proteins and modulate immune response	
	P09411	phosphoglycerate kinase 1	Pgk1	catalyze the reversible transfer of a phosphate group between 1,3-BPG and ADP	
	P48774	glutathione S-transferase Mu 5	Gstm5	catalyze the conjugation of GSH to electrophilic compounds such as toxin and ROS	
	P70290	55 kDa erythrocyte membrane protein	Mpp1	facilitate the exchange of Cl- and HCO2- across the erythrocyte membrane	
	P07901	heat shock protein HSP 90-alpha	Hsp90aa1	involved in protein folding, stability and trafficking	O
	P54116	stomatin	Stom	maintain mechanical stability of erythrocyte membrane	
	O89103	complement component C1q receptor	Cd93	initiate the classical pathway of complement activation	
	P28666	murinoglobulin-2	Mug2	involved in cell adhesion, cell signaling regulation and contribute to barrier function	
	P49222	protein 4.2	Epb42	maintain stability and integrity of erythrocyte membrane	
	Q8BFZ3	beta-actin-like protein 2	Actbl2	involved in assembly and regulation of actin cytoskeleton	
	Q8VDD5	myosin-9	Myh9	transport vesicles and organelles within the cell	
	P63242	eukaryotic translation initiation factor 5A-1	Eif5a	facilitate the initiation of protein translation on ribosomes	
Healthy plasma	Q14B46	rhotekin-2	Rtkn2	key regulator of actin cytoskeleton, cell signaling and cell adhesion	

A total 26 unique proteins are identified in the LPS plasma protein corona, while only one differential protein is found in healthy plasma corona. Acute phase proteins are highlighted with circles to underscore the potential presence of pro-inflammatory proteins associated with MDNPs.

### MDNP treatment improved survival in polymicrobial sepsis model

**Figure 7:**
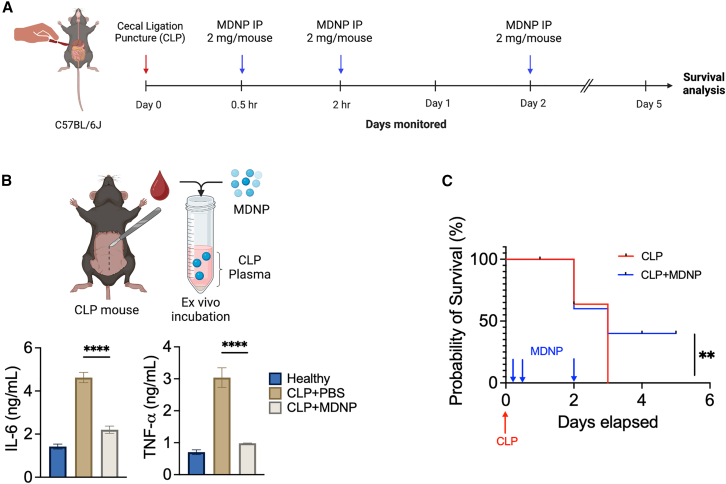
Survival effects of MDNP treatment using the severe CLP model of polymicrobial sepsis (A) Schematic timeline of the cecal ligation and puncture (CLP) survival study. C57BL/6J mice underwent CLP surgery to induce severe inflammation and polymicrobial infection and three separate doses of MDNPs were administered i.p. (0.5, 2, and 24 h time point, 2 mg/injection) to analyze survival (*n* = 5/group). Created in BioRender. Sung, J. (2025) https://BioRender.com/q03l127. (B) *Ex vivo* cytokine sequestration assay using CLP plasma following incubation with MDNPs. Created in BioRender. Sung, J. (2025) https://BioRender.com/t95w854. (C) Kaplan-Meier curves and a log rank test was performed to compare the survival rates. ∗∗*p* < 0.01. Data are expressed as mean ± SD (*n* = 4). ∗∗∗∗*p* < 0.0001 versus PBS control group.

The survival benefit of MDNPs were also tested in a clinically relevant severe polymicrobial cecal ligation and puncture (CLP) sepsis model (Figure 7A). Mice underwent the CLP procedure and were treated with three doses of 2 mg MDNPs, followed by monitoring for 5 days. To confirm that MDNPs could sequester pro-inflammatory cytokines in CLP plasma, we first incubated MDNPs with plasma isolated at 3 h post-CLP and observed an almost complete reduction in IL-6 and TNF-α (Figure 7B). In the survival study, MDNP-treated mice showed significant improvement in survival (40%) compared to control group (0%) (Figure 7C). Taken together, the ability of MDNPs to increase the survival of septic mice in the absence of antibiotics highlights their translational potential for treating inflammatory diseases, including sepsis.

## Discussion

A tremendous amount of research has been implemented to enhance the clinical management of inflammatory diseases caused by excessive stress,^39^ infections,^40^ autoimmune disorders,^41^ or secondary diseases.^42^ Despite many clinical trials aimed at blocking specific inflammatory mediators, most have been unsuccessful due to the complexity and multifactorial nature of inflammatory diseases. This highlights the need for multimodal approaches that target multiple inflammatory mediators simultaneously to improve treatment outcomes. The core pathophysiology of sepsis is known to involve the disruption of the finely tuned balance between inflammatory and anti-inflammatory immune responses.^43^ Recognition of pathogen- or damage-associated molecular patterns (PAMPs/DAMPs) by innate immune cells functions as the trigger to initiate inflammatory cell signaling and subsequent cytokine release, leading to multiorgan damage, and death. Notably, studies have shown that increased plasma IL-6 and TNF-α levels in sepsis patients are correlated with mortality.^44^^,^^45^^,^^46^ Our MDNPs efficiently reduced systemic pro-inflammatory cytokines including IL-6, TNF-α, among others (Figures 4E, 6G, and 7B), while simultaneously neutralizing additional pro-inflammatory mediators (Figure 6D). Strategies for reducing pro-inflammatory mediators include conventional adsorption resins like CytoSorb, or more sophisticated membrane-coated nanoparticles,^18^ telodendrimer nanotraps,^47^ or abiotic histone-capturing hydrogel nanoparticles.^48^ Although these approaches have shown success in certain cases, the complex structural composition and manufacturing processes may hinder rapid development. In contrast, we developed MDNPs, isolated from maca root, as a novel approach to mitigate broad pro-inflammatory responses from abundant and readily available plant material, facilitating convenient access and long-term storage potential (Figure 1). We further demonstrated their ability to reduce organ damage and improve survival as reflected in two representative and lethal sepsis models (Figures 5A, 5H, and 7C) through the formation of a multimodal protein corona (Figure 6).

Sepsis endotypes include hyperinflammatory (early) and immunosuppressive (late) states.^49^ A clinical study indicated that early-identified sepsis is associated clinically with higher mortality than late-identified sepsis.^50^ Thus, achieving control over the cytokine storm holds great potential to improve patient survival. Modes of cytokine sequestration have generally relied on charge-based interactions, where most pro-inflammatory cytokines are negatively charged, and anti-inflammatory cytokines are positively charged at neutral pH.^51^ Interestingly, the zeta potential of MDNPs were −9.6 mV (Figure 1E), yet cytokines like IL-6 (pI 6.96) and TNF-α (pI 5.01) were strongly sequestered, but IL-1β (pI 4.96) and IFN-γ (pI 8.25) were not (Figures 2K, 2L, and S6). These differences suggest the possibility of a combination of forces driving the binding of cytokines to the surface of MDNPs. Our lipidomics analysis of MDNPs (Figures 1H and 1I) showed the presence of multiple charged or ionizable lipid species like Cer (Phyto), HexCer (Phyto), FFA, LPC, and PC (Figure S2). Therefore, it is possible that a combination of local charge and hydrophobic interactions are contributing to the apparently selective sequestering of specific pro-inflammatory cytokines. Although we have not fully elaborated how MDNPs facilitate a selective binding interaction for certain cytokines, which is a limitation of our study, our results provide the basis for future studies to investigate the role of specific lipid components and their ability to alter binding interactions. Also, while our study demonstrates promising safety and therapeutic effects with the 2 mg/dose administration, future studies that investigate the impact of MDNP dose level and frequency will yield valuable information regarding the potential therapeutic enhancements or long-term safety implications, which could be particularly relevant for chronic conditions requiring sustained therapeutic intervention.

Upon exposure to biological fluids, nanoparticles dynamically adsorb biomolecules onto their surface, forming a protein corona that dictates how nanoparticles interact with host cells. These interactions can alter both drug delivery efficiency^52^ and biological function.^53^ The analysis of MDNP protein coronas revealed significant differences between MDNP-H and MDNP-LPS groups, highlighting that unique protein fingerprints are associated with MDNPs under inflammatory conditions. This is consistent with the concept of the “personalized protein corona,” which demonstrates that unique protein fingerprints are formed on the surface of nanoparticles as a function of disease state.^28^^,^^54^ The ability of MDNPs to bind cytokines in LPS- or CLP-treated mouse plasma confirmed its ability to directly modulate inflammatory responses in ongoing disease settings (Figures 6G and 7B). An unexpected finding from our proteomics analysis was the identification of multiple pro-inflammatory APPs being upregulated in the coronas of MDNP-LPS like Hp, SerpinA3N, and Saa1 (Figure 6D). Hp activates immune cells, promotes secretion of pro-inflammatory cytokines, and interacts with complement system, potentially intensifying inflammatory responses.^55^ SerpinA3N is involved in regulating inflammatory responses by controlling protease activity to prevent excessive tissue damage, but it is upregulated under inflammatory conditions.^56^ Saa1 plays direct role in recruiting immune cells and modulating pro-inflammatory cytokines.^57^ Supporting that MDNPs neutralized the pro-inflammatory functions of corona constituents, MDNP-LPS was cultured with BMDMs, and no significant immune activation was measured when evaluating IL-6 or TNF-α secretions (Figure S11). These results highlighted that MDNPs formed multimodal protein corona under inflammatory conditions that could mitigate aberrant immune activation caused by individual components, representing a novel approach to the treatment of inflammatory diseases.

Here, we demonstrated that MDNPs sequestered and neutralized multiple pro-inflammatory cytokines and APPs in their protein corona to reveal a highly effective anti-inflammatory PDNP-based therapeutic strategy for managing severe inflammatory responses. Our data indicate that MDNPs exhibit negligible toxicity, are efficiently internalized by macrophages, display immunomodulatory activities, and demonstrate significant therapeutic efficacy. Administration of MDNPs *in vivo* effectively reduced pro-inflammatory cytokines and promoted improved survival in two representative sepsis mouse models, LPS-induced endotoxemia and CLP polymicrobial sepsis. This research underscores the broad therapeutic potential of the MDNPs, which leverages a unique and never-previously-examined mechanism of action through the formation of a multimodal protein corona that effectively binds and neutralizes pro-inflammatory cytokines and APPs from propagating systemic inflammation.

## Materials and methods

### Isolation, purification, and characterization of MDNPs

A fine ground organic powder of 100% *L. meyenii* Walp (maca) (family: Brassicaceae, order: Brassicas) was purchased from an organic producer, Happy Andes (https://www.happyandes-usa.com/). Maca powder (30 g) was mixed in 400 mL of autoclaved deionized water and stirred for 12 h at RT to make maca juice. The obtained maca juice was transferred to 50 mL tubes and centrifuged at 3,000 × g for 20 min at 20°C, and then the supernatant was centrifuged again in new 50 mL tubes at 10,000 × g for 2 h at 20°C to remove small and large fibers. Next, the clear light brown colored supernatant was collected in 70 mL polycarbonate ultracentrifuge bottles (Beckman Coulter, Brea, CA) and was ultracentrifuged with using a 45 Ti rotor (Beckman Coulter) at 150,000 × g for 2 h at 4°C. Then, the collected pellets were suspended in 2 mL of phosphate-buffered saline (PBS) through dispersion with an ultrasonic processor. PBS-suspended maca pellets were transferred to a sucrose gradient (8%, 20%, 35% [g/v]) and ultracentrifuged at 150,000 × g for 2 h at 4°C, and then the cloudy band between 8% and 20% sucrose was collected as previously described.^30^ The concentration of MDNPs was measured using a Bio-Rad quantification assay. The quantified MDNPs were stored at 4°C. The size and zeta potential of MDNPs were measured by dynamic light scattering (DLS) using a Zetasizer Nano ZSP (Malvern, UK) and Nanoparticle Tracking Analysis NS300 (NTA) (Malvern, UK). MDNP stability was evaluated under various storage temperatures (RT, 4°C, −20°C, and −80°C) in PBS. In addition, its stability was tested in cell culture medium and mouse plasma at both RT and 37°C to assess behavior under biologically relevant conditions. TEM images of MDNPs were acquired using a formvar-coated copper grid and staining using 1% uranyl acetate. Differential Scanning Calorimetry (DSC) 2500 (TA Instruments, New Castle, DE) was used to analyze the temperature and heat flow associated with thermal transition in the MDNPs. Sample (5 μL) in liquid form was added into a hermetic aluminum pan and sealed with metal lid (DSC Consumables, Austin, MN) with Tzero sample press kit (TA Instruments). The following thermal procedure was used to scan: ramp 2 °C/min from 25°C to 80°C. The heat flow transition was analyzed on a plot by thermal advantage (TA) analysis software Trios (version 5.4.0.300). For lyophilization, frozen MDNPs in 1.5 mL tubes with holes on top of screw caps were placed in a benchtop freeze-dryer (Labconco, Kansas City, MO) overnight. Completely dried samples were collected and reconstituted in PBS for stability testing.

### Lipid extraction and composition analysis

A total lipid extract was prepared using a modified methyl-tert-butyl ether (MTBE) lipid extraction protocol.^58^ Briefly, 400 μL of cold methanol and 10 μL of internal standard mixture (EquiSPLASH lipidomix) were added to each sample followed by incubation at 4°C, and shaking at 650 RPM for 15 min. Next, 500 μL of cold MTBE was added followed by incubation at 4°C for 1 h with shaking at 650 RPM. Cold water (500 μL) was added slowly and resulting extract was maintained at 4°C, with shaking at 650 RPM for 15 min. Phase separation was completed by centrifugation at 8,000 RPM for 8 min at 4°C. The upper, organic phase was removed and set aside on ice. The bottom, aqueous phase was re-extracted with 200 μL of MTBE, followed by 15 min of incubation at 4°C with shaking at 650 RPM. Phase separation was completed by centrifugation at 8,000 RPM for 8 min at 4°C. The organic extract was dried under a steady stream of nitrogen at 30°C. The recovered lipids were reconstituted in 200 μL of chloroform:methanol (1:1, v/v) containing 200 μM of butylated hydroxytoluene. Prior to analysis, samples were further diluted with acetonitrile:isopropanol:water (1:2:1, v/v/v). The total lipid extract was analyzed by liquid chromatography coupled to high-resolution tandem mass spectrometry (LC-MS/MS). The LC-MS/MS analyses were performed on an Agilent 1290 Infinity LC coupled to an Agilent 6560 Quadrupole Time-of-Flight (Q-TOF) mass spectrometer. The separation was achieved using a C18 CSH (1.7 μm; 2.1 × 100 mm) column (Waters Corporation, Milford, MA, USA). Mobile phase A was 10 mM ammonium formate with 0.1% formic acid in water/acetonitrile (40:60, v/v) and mobile phase B was 10 mM ammonium formate with 0.1% formic acid in acetonitrile/isopropanol (10:90, v/v). The gradient was ramped from 40% to 43% B in 1 min, ramped to 50% in 0.1 min, ramped to 54% B in 4.9 min, ramped to 70% in 0.1 min, and ramped to 99% B in 2.9 min. The gradient was returned to initial conditions in 0.5 min and held for 1.6 min for column equilibration. The flow rate was 0.4 mL/min. The column was maintained at 55°C and the auto-sampler was kept at 5°C. A 2 μL injection was used for all samples. LC-MS data from the iterative MS/MS workflow was analyzed for lipid identification via Agilent’s Lipid Annotator (version 1.0). Positive and negative ion mode adducts included [M + H]^+^, [M+Na]^+^, [M + NH_4_]^+^, [M-H]^−^, and [M + CH_3_CO_2_]^−^, respectively. The LC-MS data from the MS^1^ workflow were processed using Agilent’s MassHunter Profinder (version 10.0).

### Characterization of MDNP protein composition and protein corona fingerprints in healthy and LPS plasma

To form the MDNP protein corona, purified MDNPs were incubated with plasma isolated from healthy and LPS-treated C57BL/6J mice for 4 h at 37°C. As described in our previous publication, MDNP bound to proteins were washed and centrifuged 3 times at 13,000 × g, 4°C with 1× cold PBS.^28^ Then it was evaluated using the following protocol. Prepared samples were lysed in a lysis buffer containing 5% sodium dodecyl sulfate (Sigma, St. Louis, MO), 50 mM triethylammonium bicarbonate (1 M, pH 8.0) (Sigma, 7408). Proteins were extracted and digested using S-trap micro columns (ProtiFi, Farmingdale, NY). The eluted peptides from the S-trap column were dried, and peptide concentration was determined using a BCA assay kit (Thermo Fisher Scientific, 23275), after reconstitution in 0.1% formic acid. All tryptic peptides were separated on a nanoACQUITY Ultra-Performance Liquid Chromatography analytical column (BEH130 C18, 1.7 μm, 75 μm × 200 mm; Waters Corporation) over a 185-min linear acetonitrile gradient with 0.1% formic acid on a nanoACQUITY Ultra-Performance Liquid Chromatography system (Waters Corporation) and analyzed on a coupled Orbitrap Fusion Lumos Tribrid mass spectrometer (Thermo Fisher Scientific, San Jose, CA). Full scans were acquired at 240,000 resolutions, and precursors were fragmented by high-energy collisional dissociation at 35% for up to 3 s. MS/MS raw files were processed using Thermo Proteome Discoverer (PD, version 3.0.0.757) with the Sequest HT search engine against the plant metagenome database and UniProt mouse reference proteome. Trypsin was used with a maximum of two missed cleavages, and peptide lengths were restricted to 6–144 amino acids. Label-free quantification was performed using the Minora feature detector. Protein identification was filtered at a 1% false discovery rate across peptide-spectrum matches (PSM), peptide, and protein levels using the Percolator algorithm in PD. Exported protein abundances were analyzed with Perseus software (version 1.6.14.0), with further filtering to include only proteins identified without missing values across all samples. The quantitative protein data were log_2_ transformed and further normalized using median centering. Two-tailed student’s t test (adjusted *p* value <0.05) was applied to determine the differentially expressed proteins. Plant data was analyzed with library from UniProt and the Plant Proteome Database (Lepidium *meyenii*: taxonomy ID 153348, entry: 92 sequences; Plant metagenome: taxonomy ID 1297885, entry: 27312 sequences; Zingiberales: taxonomy ID 4618, entry: 357900). IPA (QIAGEN) was utilized to identify canonical pathways, biological function, upstream regulators, and disease association.

### Cell culture

The generation of BMDMs followed a previously published method.^59^ As established, the tibia and femur from a C57BL/6J mouse was isolated by removing bulk muscles and connective tissues. RPMI media(supplemented with L-glutamine, penicillin [100 units/mL], streptomycin [100 μg/mL], 10% heat-inactivated fetal bovine serum [FBS], and 20% L929 cell-conditioned media) -drawn needles were inserted into bones to flush the marrow into a 10-cm Petri dish. The collected bone marrow was filtered to grow in uncoated 10-cm cell culture plates. BMDMs were cultured in RPMI media conditioned with L929 at 37°C, 5% CO_2._ The media was replaced every 3 days. On days 8–10, BMDMs were lifted using Versene (Gibco, Grand Island, NY), to be used for subsequent experiments. Trypan blue solution was used to determine the cell number and viability with an EVE Automated Cell Counter (NanoEntek, Waltham, MA). RAW-blue cells were also cultured to confluency in 75-cm^2^ flasks in identical incubation condition with Dulbecco’s modified Eagle’s medium (DMEM) supplemented with penicillin (100 U/mL), streptomycin (100 U/mL), and heat inactivated FBS (10%).

### Mice

Male C57BL/6 (6–8 weeks old) purchased from the Jackson Laboratory (Bar Harbor, ME) were maintained in cages at ambient temperature, 55% relative humidity, and under a 12 h dark/light cycle. LPS-induced endotoxemia model: mice were challenged with 5 mg/kg LPS i.p. prior to treatment with two doses of 2 mg MDNPs at 30 min and 2 h via both i.p. and i.v. injections. CLP model: mice were lightly anesthetized with a mixture of ketamine (75 mg/kg) and xylazine (15 mg/kg) at 1:1 ratio. After abdominal fur was removed, a small incision was made to expose the cecum. The cecum was then ligated with a silk suture and perforated with a 19-gauge needle. A small amount of feces was extruded by gently squeezing the cecum; the cecum was replaced, and the abdomen was sutured after the bowel was repositioned. Experimental groups were i.p-injected with three doses of 2 mg MDNPs at 30 min, 2 h, and 24 h time points. The selection of the appropriate dose was based on well-established tolerability for multiples nanoparticle administration discussed in literatures.^60^^,^^61^ All experiments were performed in compliance with the protocol by the University of Maryland, Baltimore Institutional Animal Care and Use Committee as well as the Animal Research: Reporting of *In Vivo* Experiments (ARRIVE) guidelines.

### Cytotoxicity assay

To measure the cytotoxicity of MDNPs, a propidium iodide (PI)/annexin V-FITC apoptosis assay was conducted using BMDMs. The assay utilizes PI staining to distinguish between early and late-stage apoptotic cells. Fluorescein isothiocyanate (FITC) labeling allow for the visualization of annexin V-bound cells using flow cytometry. Cultured BMDMs at a density of 1.0 × 10^5^ per well were treated with MDNPs at 1 mg/mL for 8 h, then the cells were harvested with Versene after washing with PBS. Collected cells were centrifuged at 500 × g for 5 min, and cell pellets were resuspended in 1× binding buffer. Cell suspension (100 μL) in flow cytometry tubes were added with annexin V-FITC and PI to be analyzed by flow cytometry. The data were analyzed by De Novo Software FCS Express 7 (Dotmatics, Boston, MA).

### *In vitro* cellular internalization

BMDMs were seeded overnight in Falcon Culture slides at a density of 0.5 × 10^5^ per well. Cy5.5 was incubated with MDNPs under shaking for 30 min in the dark to label the MDNPs and excessive Cy5.5 dye was removed by 20 min centrifugation at 13,000 × g prior to use. Then, 50 μL of Cy5.5-labled MDNPs were added to 0.5 mL of culture medium and incubated with cells for 0, 4, and 8 h. Negative control, untreated cells were used to establish the 0 h time point. At the determined time points, cells were washed with PBS three times, fixed with 4% paraformaldehyde (PFA) for 10 min, and dehydrated with acetone for 5 min at −20°C. After blocking the culture with 1% BSA in 1× PBS 30 min, the cells were washed again with 1× PBS and treated with 100 μL of FITC-labeled phalloidin (1:50 dilution in PBS) for 30 min to stain F-actin. The cells were then washed two times with 1× PBS and dried in the dark condition and glass cover slips were mounted with mounting medium containing 4′,6-diamidino-2-phenylindole (DAPI) after carefully removing the plastic chambers. The final fluorescence images were captured using an Olympus fluorescence microscope (Tokyo, Japan) equipped with Hamamatsu Digital Camera ORCA-03G and Nikon software was used to analyze the image data.

### Flow cytometric phenotyping of MDNP-treated macrophages

BMDMs were seeded at a density of 1.0 × 10^5^ per well were in a sterile 24-well plate. Cells were treated with 100 ng/mL of LPS in complete media for 24 h to induce inflammation. Then, 100 μg/mL and 200 μg/mL MDNPs were treated for 8 h. Cells were resuspended in MACS buffer (PBS pH 7.2 supplemented with 1% FBS and 0.4% 0.5 M EDTA, Quality Biological, Gaithersburg, MD) and transferred to the flow cytometry tubes. FcR blocking (CD16/32, BioLegend, San Diego, CA) was performed and cells were stained with following surface marker antibodies: live/dead fixable green, F4/80, CD11b, MHC II, CD206, CD80, and CD86 (BioLegend). Samples were analyzed using Cytek Aurora 3 (Fremont, CA). FCS Expression 7 Flow Cytometry De Novo Software was used for flow data processing. Mean fluorescence intensity (MFI) was generated by GraphPad Prism Software 10.1.1.

### *In vitro* pro-inflammatory cytokine sequestration and effects on NF-κB activity

Anti-inflammatory properties of MDNP were investigated in two ways: prophylactic and therapeutic. For prophylactic treatment, BMDMs at a density of 1.0 × 10^5^ per well were treated with 100 μg/mL MDNPs for 8 h and 100 ng/mL of LPS in complete media in 24-well plates (Corning, Corning, NY) were incubated for 4 h. For therapeutic study, BMDMs in identical culture condition as mentioned previously were treated with 100 ng/mL of LPS for 4 h and 100 μg/mL of MDNPs were subsequently added and incubated for 8 h. Supernatants were then collected to measure the reduction of pro-inflammatory cytokines including IL-6, TNF-α, IL-1β, and IFN-γ by enzyme-linked immunosorbent assay (ELISA) (BioLegend). We also assessed the ability of MDNPs to modulate cytokine levels in the absence of cells. The supernatants from another set of LPS-treated BMDMs were collected and incubated with 100 μg/mL of MDNPs in a time dependent manner (0, 0.5, 1, 4, 8, 12, and 24 h). To evaluate whether disrupting MDNP structure affects its ability to sequester pro-inflammatory cytokines, we reformulated MDNPs using the thin-film hydration method with additional components such as DSPE-PEG600 and cholesterol. MDNPs were first dissolved in a chloroform:methanol mixture (2:1 v/v) and subjected to rotary evaporation (Model I-300, Buchi, Switzerland) at 37°C, 100 mBar, and 280 RPM for 1 h. DSPE-PEG600 or cholesterol was then incorporated at varying concentrations (5%, 10%, and 20%) to generate distinct restructured lipid nanoparticles. Following rehydration, the nanoparticles were extruded through a 0.4 μm polycarbonate membrane using an Avanti Mini Extruder (Birmingham, AL). Lastly, we confirmed the direct ability of MDNPs to sequester IL-6 by incubating 5 ng/mL of IL-6 recombinant protein in DMEM with 100 μg/mL of MDNPs for 8 h. Remaining IL-6 levels were measured using ELISA.

An NF-κB reporter cell line, RAW-Blue, was used to determine the ability of MDNPs to alter NF-κB activity due to LPS stimulation. The cells were plated at a density of 1.0 × 10^5^ per well and were stimulated with LPS for 4 h and treated with 100 μg/mL MDNPs for 8 h. Then, QUANTI-Blue was added to the collected supernatant in flat bottom 96-well plate. It was incubated at 37°C for 1 h, and then secreted embryonic alkaline phosphatase (SEAP) reporter was detected using a spectrophotometer at 620–655 nm. Poly(lactic-co-glycolic acid) (PLGA) particles of similar size as MDNPs were used as a control and fabricated using a microfluidics device as we previously described.^62^ The size and zeta potential of the PLGA was measured 163.8 nm and −39.3 mV, respectively.

### *Ex vivo* cytokine sequestration assay

To evaluate the cytokine sequestration ability of MDNPs in mouse plasma *ex vivo*, 100 μg/mL of MDNPs in PBS was prepared and added to 100 μL of diluted LPS-treated or CLP mouse plasma to ultracentrifuge tubes. The mixture tubes were incubated at 37°C for 8 h. After incubation, samples were centrifuged at 2,000 × g for 10 min to pellet the cytokine bound MDNPs. Then the supernatant was collected to measure IL-6 and TNF-α levels using ELISA (BioLegend).

### *In vivo* biodistribution and anti-inflammatory effect following LPS challenge

The *in vivo* biodistribution study was performed by i.p. or i.v. injection of MDNPs that were labeled with the near-infrared fluorescent Cy5.5 dye. Mice were administered 2 mg of Cy5.5-MDNPs twice (0.5 and 2 h time point)-post LPS challenge (5 mg/kg) and euthanized after 4 h to collect blood by cardiac puncture and organs including the liver, lung, spleen, heart, and kidneys. The fluorescence of the organs was imaged using an IVIS with emission (720 nm) upon laser excitation (675 nm). Blood samples were centrifuged at 1,000 × g for 20 min at 4°C in microtainer capillary blood collection plasma tubes. The plasma samples were used to measure the alteration of systematic cytokines levels using a Luminex MAGPIX System (Luminex, Austin, TX). The 7-plex panel included murine IL-6, TNF-α, MCP-1, IL-10, IL-1β, IFN-β, and IFN-γ. Blood biochemistry was also measured for alanine aminotransferase (ATL/SGPT), aspartate aminotransferase (AST/SGOT), creatine (CREA), BUN, TP, and globulin, and albumin (VRL Animal Health Diagnostics, Gaithersburg, MD). The collected organs were fixed in 4% formalin immediately after euthanasia and paraffin-embedded for sectioning. H&E staining was performed using standard procedures by the Pathology Biorepository Shared Services Core at the University of Maryland, Baltimore. The level of injury scores was calculated based on the following scoring criteria: score 0, no damage; score 1, >10% slight inflammation; score 2, 10%–25% mild; score 3, 26%–50% moderate; score 4, 51%–75% severe; score 5, >75% necrosis.

### *In vivo* sepsis survival study

The *in vivo* survival study was carried out using two representative sepsis models: a lethal LPS-induced endotoxemia and CLP polymicrobial mouse model. For endotoxemia, C57BL/6J mice were challenged with 20 mg/kg LPS and two doses of MDNPs (2 mg) were administered via i.p. injection. For CLP model, the cecum of anesthetized C57BL/6J mice was perforated to release fecal materials into peritoneal cavity to generate an exacerbated polymicrobial infection. Three doses of MDNPs (2 mg/injection) were administered via i.p. route. Survival for each model was evaluated over the course of 5 days. Body temperature was also recorded every day for CLP mice.

### Statistical analysis

Data evaluation was performed using GraphPad Prism Software 10.1.1 (San Diego, CA). One-way ANOVA and t test for unpaired data were employed to evaluate statistical significance along with Tukey’s multiple comparison test. Kaplan-Meier survival curve and statistical significance of mouse survival were determined with a long-rank (Mantel-Cox) X^2^ test. Significant differences were indicated as ∗*p* < 0.05, ∗∗*p* < 0.01, ∗∗∗*p* < 0.001, and ∗∗∗*p* < 0.0001, related to control unless otherwise stated.

## Data and code availability

All lipidomics and proteomics raw data are included in the supporting information as Tables S1 and S3, respectively. Additional datasets supporting the findings are available upon reasonable request from the corresponding author.

## Acknowledgments

This work was supported by the 10.13039/100000057National Institute of General Medical Sciences of the 10.13039/100000002National Institutes of Health under award number R35GM142752 awarded to R.M.P. A.L.C. was supported by a Pharmaceutical Sciences (PSC) departmental fellowship. This publication was supported by funds through the Maryland Department of Health’s Cigarette Restitution Fund Program and the National Cancer Institute – Cancer Center Support Grant (CCSG) – P30CA134274; 10.13039/100017046University of Maryland School of Medicine’s and Greenebaum Comprehensive Cancer Center’s Flow Cytometry Core – Baltimore, Maryland; University of Maryland School of Medicine’s Center for Translational Research in Imaging – Baltimore, Maryland; University of Maryland School of Pharmacy Mass Spectrometry Center (SOP1841-IQB2014); and University of Maryland School of Medicine’s and School of Dentistry’s Electron Microscopy Core – Baltimore, Maryland. BioRender was used for preparing graphics. The content is solely the responsibility of the authors and does not necessarily represent the official view of the National Institutes of Health.

## Author contributions

J.J.S., conceptualization, formal analysis, investigation, methodology, visualization, writing – original draft, and writing – review & editing. J.R.S., methodology and writing – review & editing. J.D.R., investigation and methodology. S.D., investigation and methodology. A.L.C., investigation and methodology. M.M.W., formal analysis, investigation, and methodology. J.W.J., investigation, methodology, resources, and supervision. M.A.K., investigation, methodology, resources, and supervision. R.M.P., conceptualization, funding acquisition, methodology, resources, supervision, writing – original draft, and writing – review & editing.

## Declaration of interests

J.J.S. and R.M.P are inventors on a patent application that describes the isolation and use of MDNPs for the treatment of inflammatory diseases.

## Supplemental information

Document S1. Figures S1–S11, and Table S3

Table S1. Raw data for lipidomics analysis of MDNP composition, see supplemental spreadsheet

Table S3. Raw data for proteomics analysis of MDNP protein coronas with abundances, see supplemental spreadsheet

Document S2. Article plus supplemental information
