# Supplementary material for: Lipid nanoparticles from L. meyenii Walp mitigate sepsis through multimodal protein corona formation
Source: Mol Ther Methods Clin Dev. 2025 May 14;33(2):101491. doi: 10.1016/j.omtm.2025.101491 (PMC12151679; doi:10.1016/j.omtm.2025.101491)
Supplement: Document S1. Figures S1–S11, and Table S3 [file mmc1.pdf]

OMTM, Volume *33*

Supplemental information

Lipid nanoparticles from *L. meyenii* Walp mitigate sepsis through multimodal protein corona formation

Junsik J. Sung, Jacob R. Shaw, Josie D. Rezende, Shruti Dharmaraj, Andrea L. Cottingham, Mehari M. Weldemariam, Jace W. Jones, Maureen A. Kane, and Ryan M. Pearson

Table S1. Raw data for lipidomics analysis of MDNP composition. See Supplemental Spreadsheet.

Table S2. A complete list of total 297 proteins in healthy and LPS MDNP protein corona groups with abundances.

Accession	Description	Gene Symbol	Abundancies (Normalized) MDNP-H	Abundancies (Normalized) MDNP-LPS
P02088	Hemoglobin subunit beta-1	Hbb-b1	928003409.2	3714237272
Q00623	Apolipoprotein A-I	Apoa1	3011349023	3025579157
Q921I1	Serotransferrin	Tf	3057915073	2753587852
P01942	Hemoglobin subunit alpha	Hba	675840453	2318553770
Q61838	Pregnancy zone protein	Pzp	2195861248	2061772034
P07758	Alpha-1-antitrypsin 1-1	Serpina1a	1931662254	1832459953
Q8K0E8	Fibrinogen beta chain	Fgb	540827878.7	889052877
E9PV24	Fibrinogen alpha chain	Fga	502950428.1	839295237.1
Q8VCM7	Fibrinogen gamma chain	Fgg	457753607.8	765317995.2
Q91X72	Hemopexin	Hpx	784026907.8	709635248.8
P28665	Murinoglobulin-1	Mug1	656805594.4	701277798.1
P07759	Serine protease inhibitor A3K	Serpina3k	596369841.9	609733889.2
P01027	Complement C3	C3	689775810.9	594499422.8
Q00897	Alpha-1-antitrypsin 1-4	Serpina1d	582018556.6	561710987.6
P01872	Immunoglobulin heavy constant mu	Ighm	137882948.9	543261011.9
P07309	Transthyretin	Ttr	431047328.3	412884182.3
Q03734	Serine protease inhibitor A3M	Serpina3m	406690866.2	409369917.6
P23953	Carboxylesterase 1C	Ces1c	390060496.9	357685147.9
P08226	Apolipoprotein E	Apoe	211545786.9	356225089.1
Q00898	Alpha-1-antitrypsin 1-5	Serpina1e	290953911.7	306377089.4
P21614	Vitamin D-binding protein	Gc	211006832.2	220876877.1
P29699	Alpha-2-HS-glycoprotein	Ahsg	201801577.5	203711973.6
P20918	Plasminogen	Plg	179209268.2	161265881.4
O08677	Kininogen-1	Kng1	178334520.7	157387756.4
Q61147	Ceruloplasmin	Cp	141785124.6	144579755.1
P06728	Apolipoprotein A-IV	Apoa4	193198186	141411252.6
P11276	Fibronectin	Fn1	134339186.5	132805383.5
P22599	Alpha-1-antitrypsin 1-2	Serpina1b	137234440.7	130391478.5
P32261	Antithrombin-III	Serpinc1	132951486.3	114955170.1
P13020	Gelsolin	Gsn	122925696.2	112717714.4
P06909	Complement factor H	Cfh	112359866.2	108612025
Q01339	Beta-2-glycoprotein 1	Apoh	83917394.83	92021445.57
A6X935	Inter alpha-trypsin inhibitor, heavy chain 4	Itih4	49147988.3	76545100.49

P01864	Ig gamma-2A chain C region secreted form		90925504.67	72306405.48
P05366	Serum amyloid A-1 protein	Saa1	582569.0501	68004894.88
Q06890	Clusterin	Clu	51483662.66	66039080.19
Q61247	Alpha-2-antiplasmin	Serpinf2	63647997.95	62982489.38
P19221	Prothrombin	F2	64721089.22	55097791.25
P04186	Complement factor B	Cfb	45265576.45	50354823.92
Q61646	Haptoglobin	Hp	1175965.867	43515203.27
P01898	H-2 class I histocompatibility antigen, Q10 alpha chain	H2-Q10	50662046.24	42522868.18
E9Q414	Apolipoprotein B-100	Apob	23612875.86	41830665.25
P60710	Actin, cytoplasmic 1	Actb	36163516.91	38840668.6
P51885	Lumican	Lum	33451512.76	37404580.77
P01837	Immunoglobulin kappa constant	Igkc	29569277.12	36341454.81
P52430	Serum paraoxonase/arylesterase 1	Pon1	24541678.08	36114718.61
Q8BMT4	Transforming growth factor beta activator LRRC33	Nrros		35491678.1
Q61703	Inter-alpha-trypsin inhibitor heavy chain H2	Itih2	30003313.58	34773679.85
P29788	Vitronectin	Vtn	32577254.54	33474417.73
Q9DBD0	Inhibitor of carbonic anhydrase	Inhca	32459170.46	33009210.91
P04104	Keratin, type II cytoskeletal 1	Krt1	14418404.79	32569229.54
P01029	Complement C4-B	C4b	43609773.77	32020835.64
B5X0G2	Major urinary protein 17	Mup17	18463893.04	31477651.07
P33622	Apolipoprotein C-III	Apoc3	34176831.56	31117842.48
O89020	Afamin	Afm	30206702.58	31068301.53
P06684	Complement C5	C5	35253338.66	29841220.55
Q91WP6	Serine protease inhibitor A3N	Serpina3n	9115387.226	29170213.55
P97290	Plasma protease C1 inhibitor	Serping1	26434129.42	28809827.38
P00920	Carbonic anhydrase 2	Ca2	9757950.724	27971880.09
Q01279	Epidermal growth factor receptor	Egfr	30873381.51	27314963.99
P03987	Ig gamma-3 chain C region		34390879.06	25243150.62
P26262	Plasma kallikrein	Klkb1	26344380.27	25030888.74
Q9ESB3	Histidine-rich glycoprotein	Hrg	26029322.31	24775219.51
Q8CG14	Complement C1s-1 subcomponent	C1s1	60137064.73	24636430.62
P09813	Apolipoprotein A-II	Apoa2	27831505.41	24508337.48
Q61702	Inter-alpha-trypsin inhibitor heavy chain H1	Itih1	22930405.01	22799950.79
P34928	Apolipoprotein C-I	Apoc1	25204072.49	22481305.91
P06330	Ig heavy chain V region AC38 205.12		15878369.91	21571691.03
O70362	Phosphatidylinositol-glycan-specific phospholipase D	Gpld1	22655078.01	20820121.94
Q9DBB9	Carboxypeptidase N subunit 2	Cpn2	21174648.27	19600603.73
P04919	Band 3 anion transport protein	Slc4a1	3560012.489	19518362.92

P01867	Immunoglobulin heavy constant gamma 2B	Ighg2b	30395532.68	19385640.22
P01675	Ig kappa chain V-VI region XRPC 44		12354393.56	18510890.9
Q8BH35	Complement component C8 beta chain	C8b	18366062.6	17641935.99
Q9QWK4	CD5 antigen-like	Cd5l	7468381.624	17619564.27
Q9QXC1	Fetuin-B	Fetub	20412337.94	17259520.95
Q8BND5	Sulfhydryl oxidase 1	Qsox1	11781957.39	16718825.16
Q8K182	Complement component C8 alpha chain	C8a	17208559.76	16571859.85
P06683	Complement component C9	C9	15659305.13	16436359.6
Q07456	Protein AMBP	Ambp	12374958.07	15609278.38
Q61129	Complement factor I	Cfi	15558148.66	15435179.98
Q8VCG4	Complement component C8 gamma chain	C8g	15279649.9	14632890.46
P01869	Ig gamma-1 chain C region, membrane-bound form	Ighg1	52910504.46	14494589.13
Q922U2	Keratin, type II cytoskeletal 5	Krt5	4207610.432	14406270.58
Q60590	Alpha-1-acid glycoprotein 1	Orm1	14133693.05	14145322.48
Q06770	Corticosteroid-binding globulin	Serpina6	15845190.92	14088927.48
P08607	C4b-binding protein	C4bpa	14054630.25	12472498.93
P05367	Serum amyloid A-2 protein	Saa2		12117809.23
Q61171	Peroxiredoxin-2	Prdx2	3085888.231	12099945.33
P03953	Complement factor D	Cfd	12019059.35	11739969.15
P46412	Glutathione peroxidase 3	Gpx3	18460002.92	11098693.44
P02535	Keratin, type I cytoskeletal 10	Krt10	9677242.7	10915950.63
Q64726	Zinc-alpha-2-glycoprotein	Azgp1	10338349.62	10709609
P11859	Angiotensinogen	Agt	7796111.559	10355122.87
Q00724	Retinol-binding protein 4	Rbp4	5845338.528	9707910.377
Q3UV17	Keratin, type II cytoskeletal 2 oral	Krt76	3509402.221	9209172.535
P01592	Immunoglobulin J chain	Jchain	2866037.289	9103479.2
Q02105	Complement C1q subcomponent subunit C	C1qc	8399420.657	8647158.17
Q9Z1R3	Apolipoprotein M	Apom	7701827.69	8550116.051
P31532	Serum amyloid A-4 protein	Saa4	7586819.857	8415684.275
Q3TTY5	Keratin, type II cytoskeletal 2 epidermal	Krt2	4551184.715	7929576.352
P49182	Heparin cofactor 2	Serpind1	8334838.569	7270216.039
P11589	Major urinary protein 2	Mup2	3056383.124	7112354.108
P51910	Apolipoprotein D	Apod	7550189.187	7075306.394
Q5FW60	Major urinary protein 20	Mup20	2352747.672	6801889.379
Q61704	Inter-alpha-trypsin inhibitor heavy chain H3	Itih3	6604642.255	6787648.943
Q61730	Interleukin-1 receptor accessory protein	Il1rap	7482934.893	6706985.092
P42703	Leukemia inhibitory factor receptor	Lifr	5740631.518	6664111.113
P13634	Carbonic anhydrase 1	Ca1	2848827.054	6608731.101

Q9JJN5	Carboxypeptidase N catalytic chain	Cpn1	8100394.557	6459556.99
P35441	Thrombospondin-1	Thbs1	101055.5742	6411564.188
O88947	Coagulation factor X	F10	11204744.52	6302198.774
P01887	Beta-2-microglobulin	B2m	6897094.775	6141391.565
P14847	C-reactive protein	Crp	3275921.805	5828717.652
P16858	Glyceraldehyde-3-phosphate dehydrogenase	Gapdh	1017889.26	5791969.956
P70274	Selenoprotein P	Selenop	5075468.562	5714927.268
Q80YC5	Coagulation factor XII	F12	5250962.792	5281776.716
P98086	Complement C1q subcomponent subunit A	C1qa	5422527.892	5262030.37
Q9Z126	Platelet factor 4	Pf4		5059918.916
P16301	Phosphatidylcholine-sterol acyltransferase	Lcat	4408647.341	4968372.195
P14106	Complement C1q subcomponent subunit B	C1qb	4875764.818	4958952.405
O88783	Coagulation factor V	F5	2642773.202	4874498.276
Q8R121	Protein Z-dependent protease inhibitor	Serpina10	4870612.494	4802069.979
P55065	Phospholipid transfer protein	Pltp	4973443.774	4531456.258
P01727	Ig lambda-1 chain V region S43		4235723.765	4531136.166
P01655	Ig kappa chain V-III region PC 7132		2173667.333	4148828.728
Q923D2	Flavin reductase (NADPH)	Blvrb	1210426.717	4053489.672
Q61268	Apolipoprotein C-IV	Apoc4	5251213.727	4032759.88
P04945	Ig kappa chain V-VI region NQ2-6.1		645056.2321	4026117.531
Q9R098	Hepatocyte growth factor activator	Hgfac	5913123.193	3968114.69
P41317	Mannose-binding protein C	Mbl2	3274967.556	3947541.403
P01631	Ig kappa chain V-II region 26-10		8099471.584	3928048.55
P01801	Ig heavy chain V-III region J606		1864864.172	3764640.681
Q60994	Adiponectin	Adipoq	5218288.283	3592772.863
P07310	Creatine kinase M-type	Ckm	93710.93473	3509827.392
P01843	Ig lambda-1 chain C region		3421048.039	3504298.725
P01803	Ig heavy chain V region AMPC1		587200.1683	3323377.141
P04247	Myoglobin	Mb	111294.5766	3188757.356
P11680	Properdin	Cfp	4110900.538	3140100.888
P04939	Major urinary protein 3	Mup3	1602452.567	3092342.071
P97298	Pigment epithelium-derived factor	Serpinf1	2630407.051	3033494.276
P01680	Ig kappa chain V-IV region S107B		827532.5022	2963434.332
Q61508	Extracellular matrix protein 1	Ecm1	2409400.968	2826543.008
Q9Z2K1	Keratin, type I cytoskeletal 16	Krt16	385143.2046	2819454.191
Q8VED5	Keratin, type II cytoskeletal 79	Krt79	1212706.798	2776684.772
P01635	Immunoglobulin kappa chain variable 12-41	Igkv12-41	427261.8639	2736059.576
P50446	Keratin, type II cytoskeletal 6A	Krt6a		2707708.999

Q02357	Ankyrin-1	Ank1	36773.72166	2629400.818
P15508	Spectrin beta chain, erythrocytic	Sptb	506477.6171	2626512.316
P08032	Spectrin alpha chain, erythrocytic 1	Spta1	452189.6159	2593533.771
P01665	Ig kappa chain V-III region PC 7043		171570.79	2580106.965
P04918	Serum amyloid A-3 protein	Saa3	635270.9355	2457250.179
Q8BH61	Coagulation factor XIII A chain	F13a1	6117289.427	2293677.485
Q91WP0	Mannan-binding lectin serine protease 2	Masp2	2541102.696	2285064.866
P01878	Ig alpha chain C region		3224291.133	2237437.758
P10126	Elongation factor 1-alpha 1	Eef1a1	441537.694	2159080.241
P70389	Insulin-like growth factor-binding protein complex acid labile subunit	Igfals	2224727.582	2081860.873
P08071	Lactotransferrin	Ltf		2037189.124
P18528	Ig heavy chain V region 6.96		1520840.757	1955728.595
P62737	Actin, aortic smooth muscle	Acta2	488055.7673	1905757.552
P01644	Ig kappa chain V-V region HP R16.7		2311965.542	1845398.545
P62983	Ubiquitin-ribosomal protein eS31 fusion protein	Rps27a	654733.5784	1728212.099
Q8VCS0	N-acetylmuramoyl-L-alanine amidase	Pglyrp2	1968563.854	1631723.507
P09581	Macrophage colony-stimulating factor 1 receptor	Csf1r	1299863.116	1629887.006
Q61781	Keratin, type I cytoskeletal 14	Krt14	277383.4727	1589487.206
P24270	Catalase	Cat	237052.1895	1575287.396
P01638	Ig kappa chain V-V region L6		1079440.707	1533036.36
P02104	Hemoglobin subunit epsilon-Y2	Hbb-y	355490.4868	1498424.351
Q62351	Transferrin receptor protein 1	Tfrc	987638.7793	1469745.1
Q9JHH6	Carboxypeptidase B2	Cpb2	1539544.598	1450523.22
P05064	Fructose-bisphosphate aldolase A	Aldoa	183641.871	1446645.03
Q07968	Coagulation factor XIII B chain	F13b	3531441.262	1427418.032
P01639	Immunoglobulin kappa chain variable 9-120	Igkv9-120	1482319.534	1391522.029
P15327	Bisphosphoglycerate mutase	Bpgm	517656.4183	1388413.446
P01786	Ig heavy chain V region MOPC 47A		378980.9374	1346201.385
P61939	Thyroxine-binding globulin	Serpina7	1050672.157	1306869.921
P01897	H-2 class I histocompatibility antigen, L-D alpha chain	H2-L	782551.5554	1305391.625
P39039	Mannose-binding protein A	Mbl1	804163.9994	1292026.996
O70165	Ficolin-1	Fcn1	989373.7446	1289473.009
P33587	Vitamin K-dependent protein C	Proc	918418.4606	1245425.217
P01844	Ig lambda-2 chain C region	Iglc2	933514.4961	1199107.648
P11352	Glutathione peroxidase 1	Gpx1	448819.0804	1156240.245
Q02013	Aquaporin-1	Aqp1	290921.9173	1152957.361
P01787	Ig heavy chain V regions TEPC 15/S107/HPCM1/HPCM2/HPCM3		773081.5033	1152956.555
P51437	Cathelicidin antimicrobial peptide	Camp		1141140.866

P18525	Ig heavy chain V region 5-84		929417.0169	1113167.285
Q61805	Lipopolysaccharide-binding protein	Lbp		1106587.479
P06151	L-lactate dehydrogenase A chain	Ldha	83259.45611	1068558.922
Q8BPB5	EGF-containing fibulin-like extracellular matrix protein 1	Efemp1	957110.2576	1051273.401
P18524	Ig heavy chain V region RF		996843.9067	1027726.632
Q9CQW3	Vitamin K-dependent protein Z	Proz	1475605.916	1018596.468
Q8CG16	Complement C1r-A subcomponent	C1ra	1072269.264	1007713.106
P01758	Ig heavy chain V region 108A	Igh-VJ558	311409.0389	1003340.943
O35930	Platelet glycoprotein Ib alpha chain	Gp1ba	1210609.365	973056.378
P17742	Peptidyl-prolyl cis-trans isomerase A	Ppia		962190.9382
P21180	Complement C2	C2	974672.7363	915365.3758
P52480	Pyruvate kinase PKM	Pkm	66810.24807	768374.2116
P01643	Ig kappa chain V-V region MOPC 173		676756.4934	759604.6229
P11404	Fatty acid-binding protein, heart	Fabp3	49282.86044	751989.3473
Q9WVJ3	Carboxypeptidase Q	Cpq	481426.3933	745130.3249
P63017	Heat shock cognate 71 kDa protein	Hspa8	452344.8245	725191.1799
Q6IMP4	Pannexin-2	Panx2	673329.6096	714597.5646
P62827	GTP-binding nuclear protein Ran	Ran	240544.5144	709241.6649
P17751	Triosephosphate isomerase	Tpi1	59028.4323	681226.2498
P62806	Histone H4	H4c1		650320.7572
P08228	Superoxide dismutase [Cu-Zn]	Sod1	284304.0683	633104.1819
P01662	Ig kappa chain V-III region ABPC 22/PC 9245		243595.2228	598474.2129
P00687	Alpha-amylase 1	Amy1	636575.4654	593350.912
P98064	Mannan-binding lectin serine protease 1	Masp1	700603.2732	563752.9026
P01750	Ig heavy chain V region 102		714952.629	551904.1395
P10810	Monocyte differentiation antigen CD14	Cd14		545869.5645
P11672	Neutrophil gelatinase-associated lipocalin	Lcn2		535419.015
P14152	Malate dehydrogenase, cytoplasmic	Mdh1		534558.042
O88968	Transcobalamin-2	Tcn2	474797.4922	529356.3326
P10605	Cathepsin B	Ctsb	369116.9063	507950.2788
O08899	Regulator of G-protein signaling 4	Rgs4	508507.6258	499218.1259
P01642	Ig kappa chain V-V region L7	Gm10881	495826.2892	496242.8161
Q9ET66	Peptidase inhibitor 16	Pi16	95961.47752	479325.348
P35700	Peroxiredoxin-1	Prdx1	117636.8253	478144.7418
P14430	H-2 class I histocompatibility antigen, Q8 alpha chain	H2-Q8	239335.4636	469309.8116
Q08879	Fibulin-1	Fbln1	419877.2255	426677.1854
P53986	Monocarboxylate transporter 1	Slc16a1	28623.60822	412682.9054
P27661	Histone H2AX	H2ax	109172.7495	382814.2872

Q8K1I3	Secreted phosphoprotein 24	Spp2	338014.7574	374289.839
P23492	Purine nucleoside phosphorylase	Pnp		365869.5486
P48193	Protein 4.1	Epb41	103875.1831	344479.818
P06327	Ig heavy chain V region VH558 A1/A4	Gm5629	108375.0834	332725.2552
P01633	Immunoglobulin kappa chain variable 6-17	Igkv6-17	197004.8172	322070.4402
P01728	Ig lambda-2 chain V region		217489.6166	319542.4043
P04117	Fatty acid-binding protein, adipocyte	Fabp4		315778.6717
P16015	Carbonic anhydrase 3	Ca3		290487.8667
Q61330	Contactin-2	Cntn2	134956.1178	289166.3145
O55042	Alpha-synuclein	Snca	168409.0136	282600.449
P10639	Thioredoxin	Txn	114151.1231	282118.4762
P32848	Parvalbumin alpha	Pvalb		281275.3964
P70296	Phosphatidylethanolamine-binding protein 1	Pebp1		239032.0395
P01630	Ig kappa chain V-II region 7S34.1		280837.7285	220323.3254
Q3SXB8	Collectin-11	Colec11	167401.0377	220086.0044
P01902	H-2 class I histocompatibility antigen, K-D alpha chain	H2-K1	96439.86213	208128.1475
O55028	Branched-chain alpha-ketoacid dehydrogenase kinase	Bckdk	736090.4191	206778.6285
Q62009	Periostin	Postn	152431.3956	201674.5953
Q8K426	Resistin-like gamma	Retnlg	60400.40284	197528.973
P29533	Vascular cell adhesion protein 1	Vcam1	154212.4878	195456.0182
Q8R0Z6	Angiopoietin-related protein 6	Angptl6	148958.7772	194914.202
Q9QUM9	Proteasome subunit alpha type-6	Psma6	102363.4692	193835.0291
P43025	Tetranectin	Clec3b	394119.509	192169.469
Q9D358	Low molecular weight phosphotyrosine protein phosphatase	Acp1		186169.208
Q99PT1	Rho GDP-dissociation inhibitor 1	Arhgdia		185554.6829
P16294	Coagulation factor IX	F9	205489.3198	176598.7952
O08692	Neutrophilic granule protein	Ngp		173644.8302
P01660	Ig kappa chain V-III region PC 3741/TEPC 111		451258.3076	172938.9516
P26928	Hepatocyte growth factor-like protein	Mst1	220308.4434	165340.6285
P62259	14-3-3 protein epsilon	Ywhae	25740.68703	165025.5098
Q64339	Ubiquitin-like protein ISG15	Isg15		155448.3283
P01819	Ig heavy chain V region MOPC 141		111665.7851	153986.9175
P13597	Intercellular adhesion molecule 1	Icam1	35830.14385	153049.0449
Q8BK48	Pyrethroid hydrolase Ces2e	Ces2e	138648.6251	141534.1001
Q9DA19	Corepressor interacting with RBPJ 1	Cir1	143355.989	131390.7276
P09411	Phosphoglycerate kinase 1	Pgk1		128763.9831
P48774	Glutathione S-transferase Mu 5	Gstm5		128138.7795
P45700	Mannosyl-oligosaccharide 1,2-alpha-mannosidase IA	Man1a1	111866.5159	125361.5957

P70290	55 kDa erythrocyte membrane protein	Mpp1		120048.7397
O08709	Peroxiredoxin-6	Prdx6	45497.51336	117590.9447
O70435	Proteasome subunit alpha type-3	Psma3	56781.75446	111797.9731
P01674	Ig kappa chain V-III region PC 2154		180993.5702	106219.4872
P07901	Heat shock protein HSP 90-alpha	Hsp90aa1		100481.8017
P54116	Stomatin	Stom		98450.13141
P21460	Cystatin-C	Cst3	53373.02358	94348.54252
P01636	Ig kappa chain V-V region MOPC 149		68964.84947	92414.91495
P62962	Profilin-1	Pfn1	25524.30285	91376.00012
P01741	Ig heavy chain V region		165123.7841	88549.54136
P10853	Histone H2B type 1-F/J/L	H2bc11; H2bc13; H2bc15; H2bc7		85259.87042
P30412	Peptidyl-prolyl cis-trans isomerase C	Ppic	54402.95832	84604.76957
Q8CIF4	Biotinidase	Btd	72066.23784	82305.05012
O89103	Complement component C1q receptor	Cd93		75276.41839
Q05020	Apolipoprotein C-II	Apoc2	97660.29413	67462.46407
P18337	L-selectin	Sell	58937.10843	66985.2593
Q9R1P4	Proteasome subunit alpha type-1	Psma1	50646.0636	66984.96607
Q8CFG8	Complement C1s-1 subcomponent	C1s2	62510.4846	55936.92934
Q01853	Transitional endoplasmic reticulum ATPase	Vcp	15995.53715	48063.65088
P09470	Angiotensin-converting enzyme	Ace	54092.72918	36537.61521
Q9DCD0	6-phosphogluconate dehydrogenase, decarboxylating	Pgd	14439.63011	33679.13632
P63242	Eukaryotic translation initiation factor 5A-1	Eif5a		18835.75916
Q01755	T-complex protein 11	Tcp11		18424.59229
P97861	Keratin, type II cuticular Hb6	Krt86	1574186.28	
Q8K0Y2	Keratin, type I cuticular Ha3-I	Krt33a	1196179.549	
P82198	Transforming growth factor-beta-induced protein ig-h3	Tgfbi	206323.6036	
P70663	SPARC-like protein 1	Sparcl1	59116.26748	23560.18026
Q03311	Cholinesterase	Bche	17536.47352	48489.92455
Q8BU03	Periodic tryptophan protein 2 homolog	Pwp2		227138.7337
P05208	Chymotrypsin-like elastase family member 2A	Cela2a		74629.05644
Q60805	Tyrosine-protein kinase Mer	Mertk		38109.23059
P02089	Hemoglobin subunit beta-2	Hbb-b2		262087.4739
P12246	Serum amyloid P-component	Apcs		307144.909
P28666	Murinoglobulin-2	Mug2		
Q14B46	Rhotekin-2	Rtkn2	2911346.75	

Table S3. Raw data for proteomics analysis of MDNP protein coronas with abundances. See Supplemental Spreadsheet.

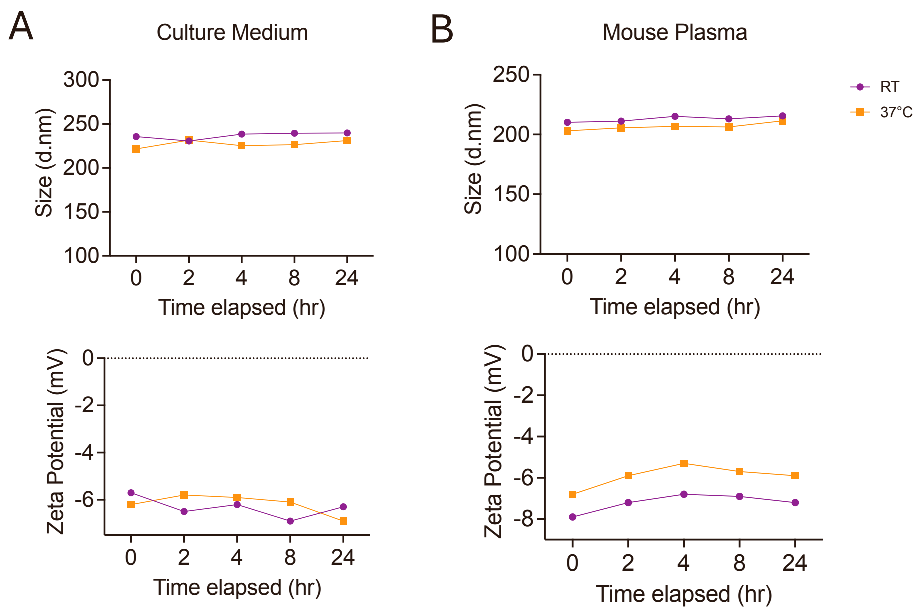

Figure S1. Stability test for MDNP in culture medium and mouse plasma. MDNPs were incubated in (A) culture medium or (B) mouse plasma at RT and 37ºC for up to 24 hrs, with continuous size and zeta potential monitoring.

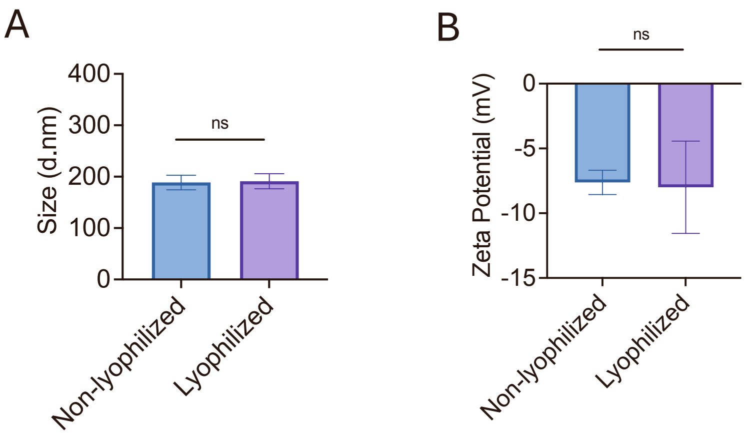

Figure S2. Stability test for lyophilized MDNPs with and without cryoprotectant. (A) Size and (B) zeta potential were measured after reconstituting lyophilized MDNPs with cryoprotectant and without cryoprotectant supplementation.

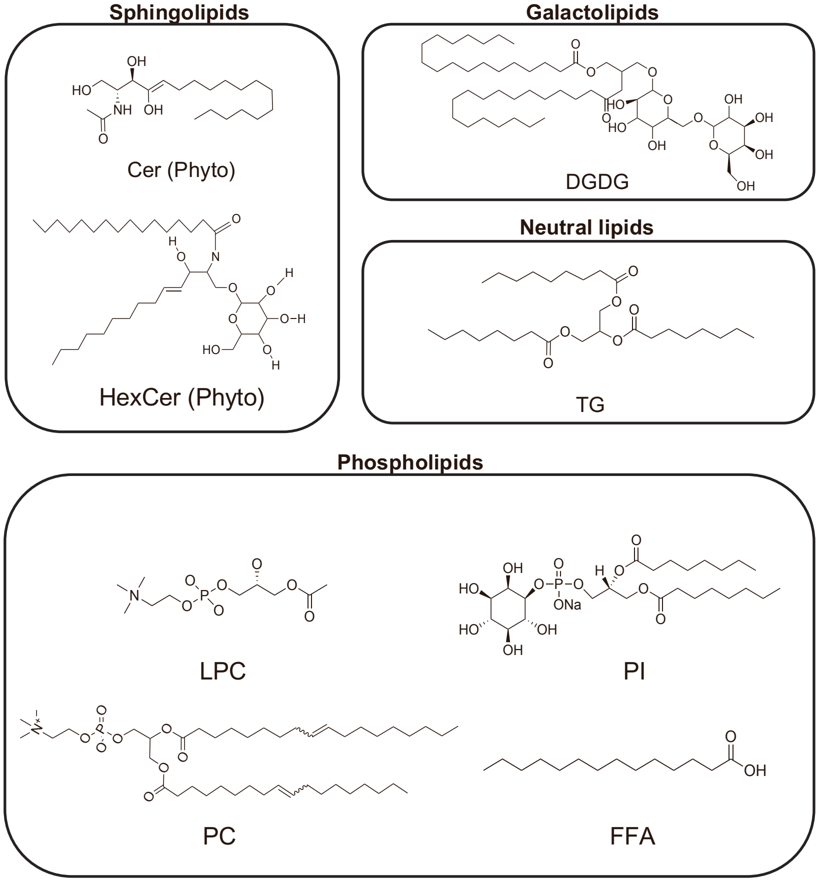

Figure S3. Chemical structure of lipid components in four distinctive lipid groups (sphingolipids, galactolipids, neutral, and phospholipids).

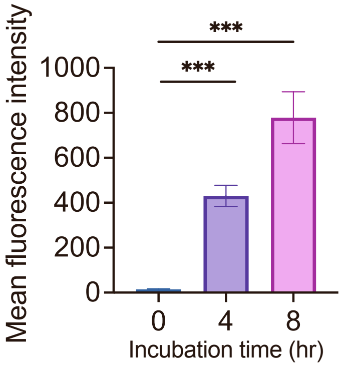

Figure S4. Mean fluorescence intensity (MFI) quantification of Cy5.5-labeled MDNP uptake in BMDMs at 0, 4, and 8 hr timepoints. ***p<0.001.

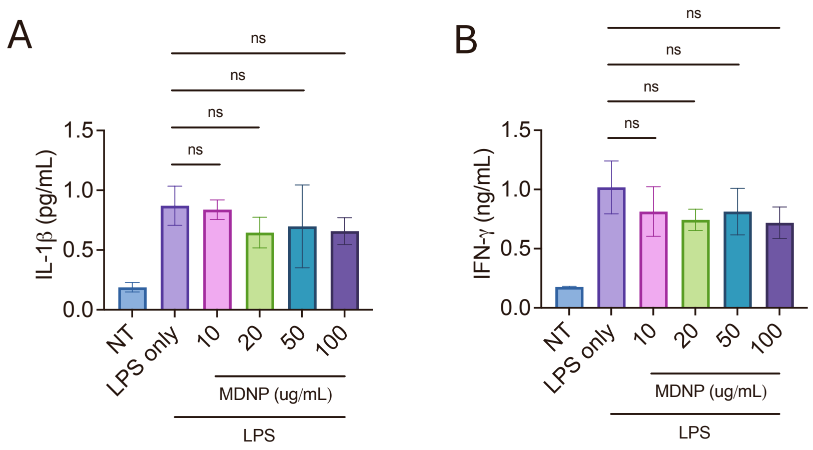

Figure S5. ELISA quantification of pro-inflammatory cytokines (A) IL-1β and (B) IFN-γ after therapeutic treatment of MDNPs *in vitro*.

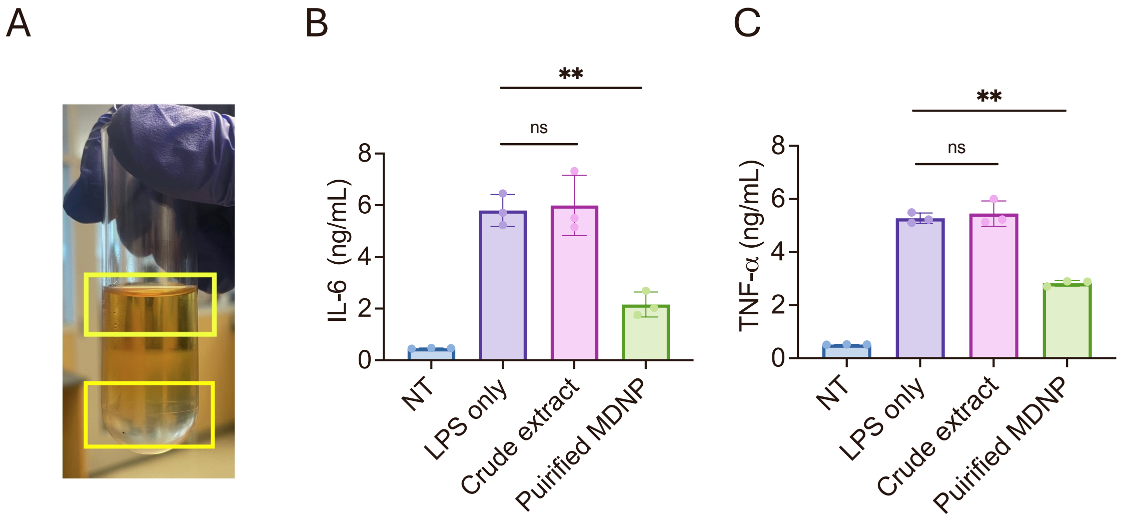

Figure S6. Loss of anti-inflammatory effect in crude extract after MDNP isolation. (A) Crude extract in yellow rectangles is the remaining solution after MDNP isolation through sucrose gradient separation. Quantification of pro-inflammatory cytokines (B) IL-6 and (C) TNF-α after BMDM treatment with MDNPs and crude extract from maca juice. **$p<0.01$.

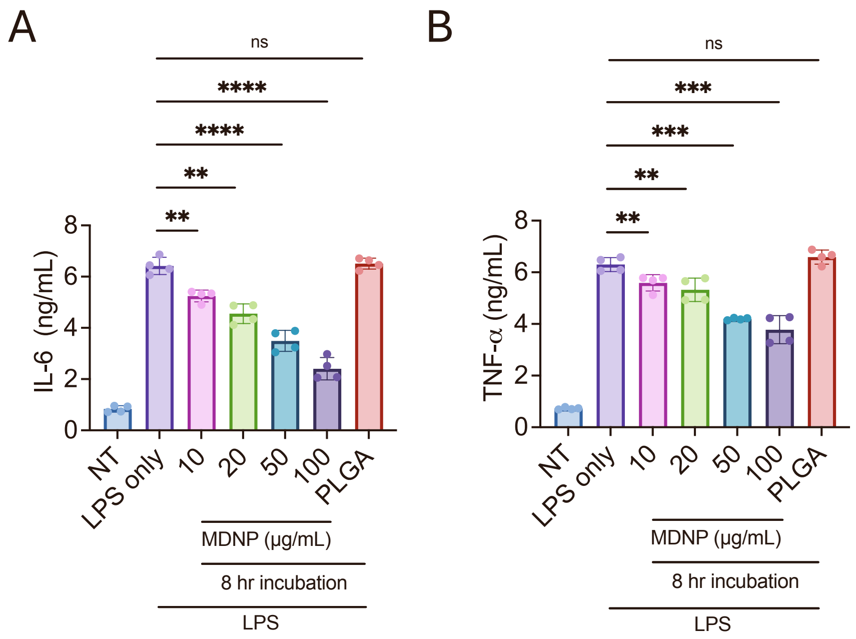

Figure S7. MDNP concentration-dependent cytokine reduction of (A) IL-6 and (B) TNF-α after 8 hr therapeutic treatment in BMDMs. **p<0.01, ***p<0.001, ****p<0.0001.

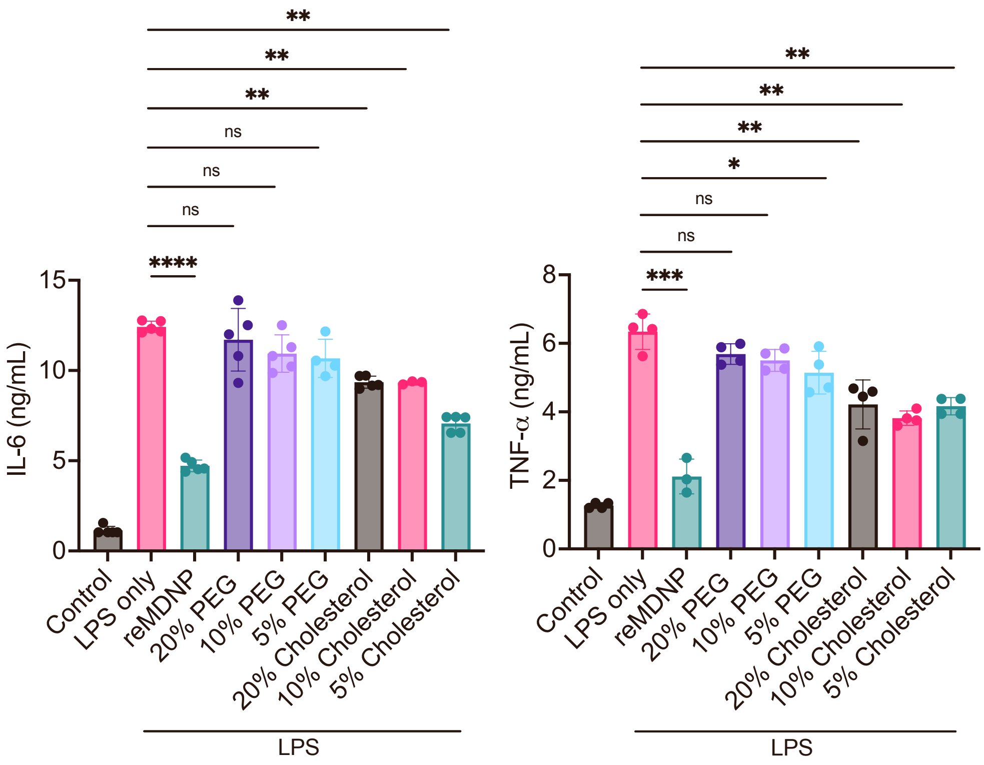

Figure S8. Restructured MDNP (reMDNP) retains its ability to sequester cytokines whereas disrupted MDNP by PEG and cholesterol exhibited partial or complete loss of function to remove pro-inflammatory cytokines, (A) IL-6 and (B) TNF-α. *p<0.05, **p<0.01, ***p<0.001, ****p<0.0001.

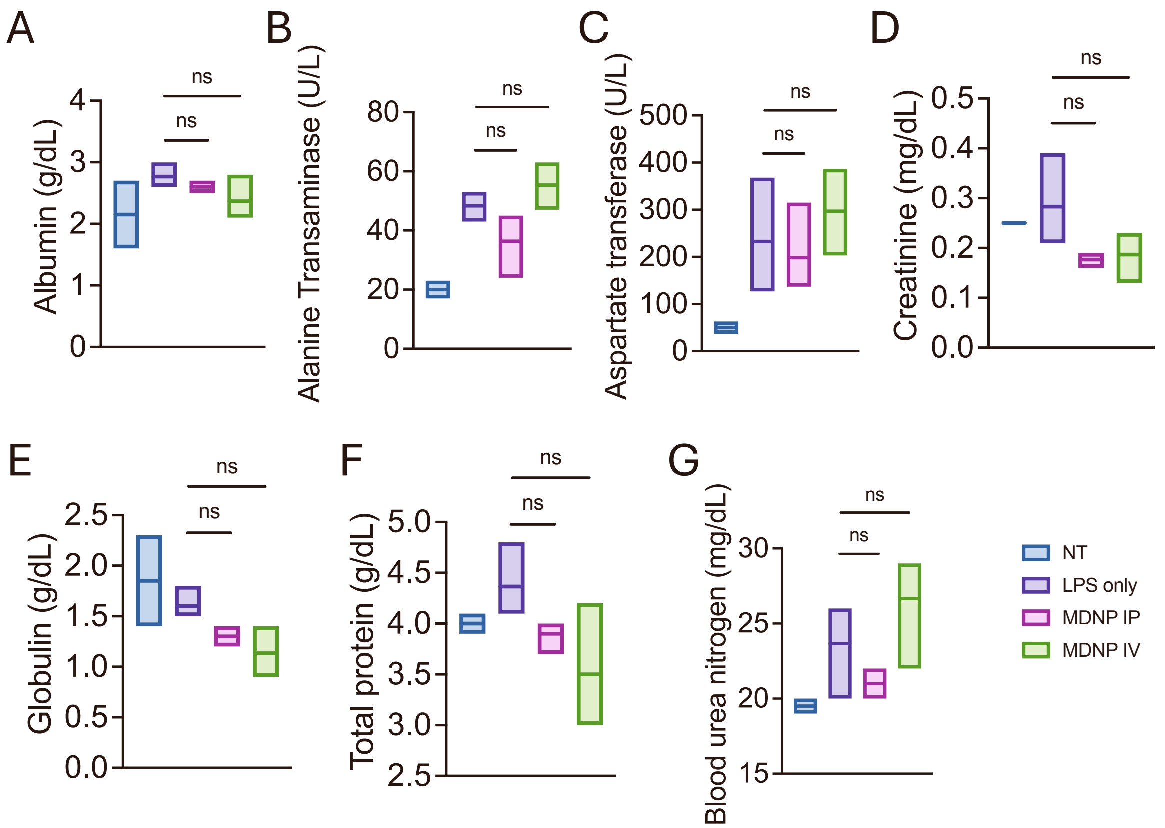

Figure S9. Blood biochemistry analysis of LPS challenged and MDNP administered mice plasma for (A) Albumin, (B) Alanine Transaminase, (C) Aspartate transferase, (D) Creatinine, (E) Globulin, (F) Total protein, and (G) Blood Urea Nitrogen. All data is expressed as mean ± SD (n=3/group).

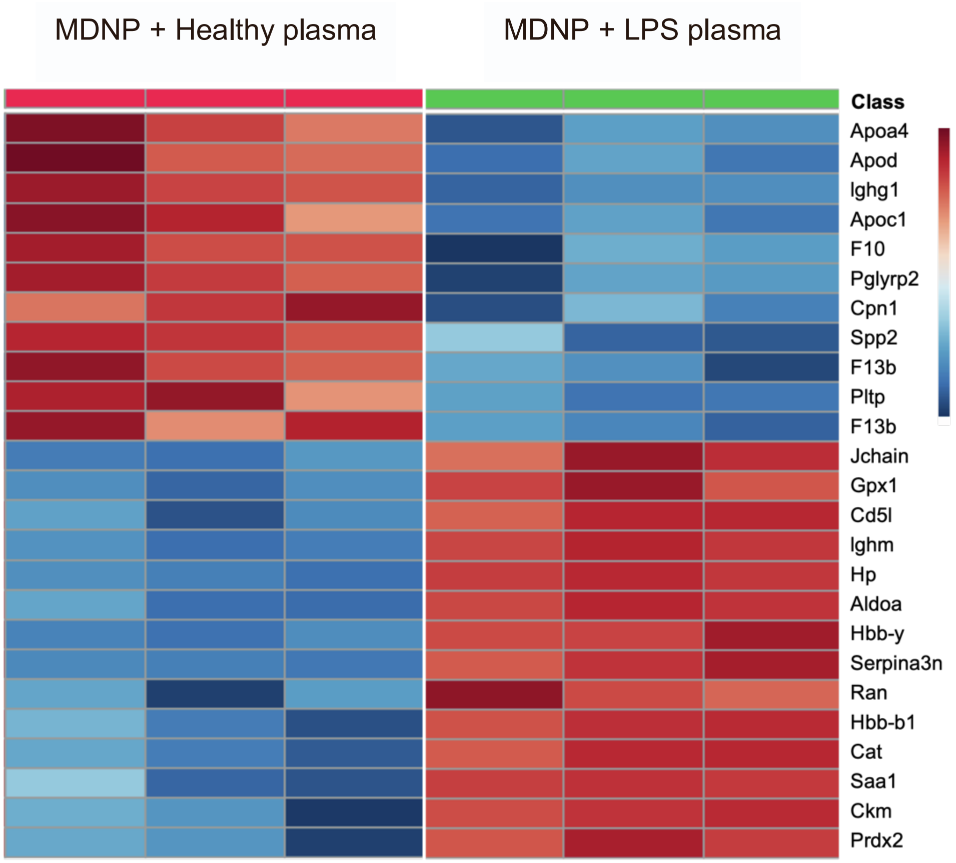

Figure S10. Top 25 proteins that are upregulated (Red) and downregulated (Blue) in each healthy and LPS plasma incubated group. N=3/group.

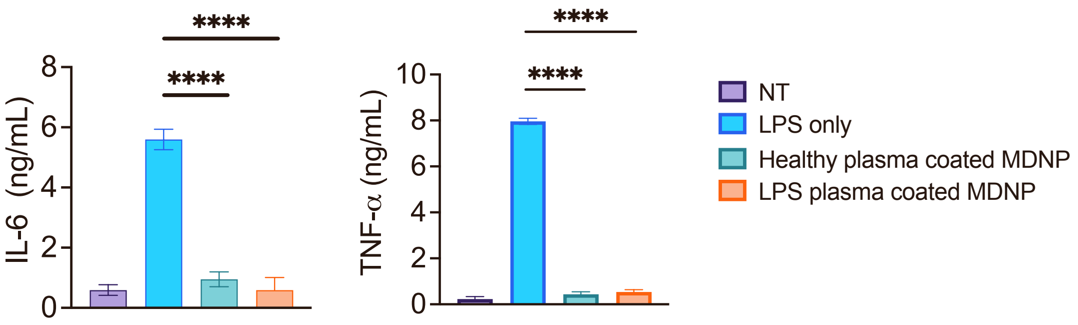

Figure S11. MDNPs coated in LPS-stimulated mouse plasma were tested to assess corona-dependent cytokine production in BMDMs. All data are expressed as means ± SD (n=3). ****p<0.0001 versus LPS only group.
